# Mononuclear Transition Metal Cymantrenecarboxylates as Precursors for Spinel-Type Manganites

**DOI:** 10.3390/molecules27031082

**Published:** 2022-02-06

**Authors:** Pavel S. Koroteev, Andrey B. Ilyukhin, Andrey V. Gavrikov, Konstantin A. Babeshkin, Nikolay N. Efimov

**Affiliations:** N.S. Kurnakov Institute of General and Inorganic Chemistry, Russian Academy of Sciences, Leninsky Prosp. 31, 119991 Moscow, Russia; ilyukhin@gmail.com (A.B.I.); penguin1990@yandex.ru (A.V.G.); bkonstantan@yandex.ru (K.A.B.); nnefimov@yandex.ru (N.N.E.)

**Keywords:** cymantrenecarboxylate, transition metals, heterometallic complexes, thermolysis, spinel, manganite, magnetic properties, single-ion magnet

## Abstract

Novel mononuclear cymantrenecarboxylate complexes of transition metals, [Co(H_2_O)_6_](CymCO_2_)_2_·4H_2_O (Cym = (η^5^-C_5_H_4_)Mn(CO)_3_) (**1**), [Ni(H_2_O)_6_](CymCO_2_)_2_·4H_2_O (**2**), [Zn(H_2_O)_6_](CymCO_2_)_2_·4H_2_O (**3**), [Co(CymCO_2_)_2_(imz)_2_] (imz = imidazole, **4**), [Co(CymCO_2_)_2_(bpy)_2_]·2PhMe (bpy = 2,2′-bipyridyl, **5**), [Ni(CymCO_2_)(bpy)_2_(H_2_O)][CymCO_2_]·0.5MePh·2H_2_O (**6**), [Cu(CymCO_2_)_2_(imz)_2_] (**7**), and [Cu(CymCO_2_)_2_(bpy)(H_2_O)] (**8**), were obtained and characterized by single-crystal X-ray analysis. Complexes **1**–**3** are isostructural. Magnetism of the Co complexes **1**, **4**, and **5** was studied; it was shown that they exhibit the properties of field-induced single-molecule magnets with magnetization reversal barriers (ΔE/k_B_) of 44, 13, and 10 K, respectively. Thermal decomposition of complexes **1**–**8** was studied by means of DSC and TGA methods. The final products of thermolysis of **1**–**6** in air, according to powder XRD data, are the pure spinel phases MMn_2_O_4_; for the cases of copper complexes, the mixtures of CuMn_2_O_4_ and CuO were found in the products.

## 1. Introduction

Initially, the formation of polynuclear heterometallic complexes of transition metals in solution was determined by means of physicochemical methods in the 1960s [1]. Subsequently, 3d-3d′-heterometallic complexes based on Schiff bases were isolated as individual compounds; however, they were not characterized by X-ray diffraction analysis [2]. Since the beginning of the 1980s, many various 3d-3d′-heterometallic complexes in which different metal ions were bound by typical polydentate ligands (Schiff bases, amino alcohols, amino phenols, amino acids), including carboxylates, were obtained and structurally characterized [3,4,5,6]. Interest in such compounds was raised by their potential unusual properties caused by the presence of metal atoms of various nature in one molecule; this made it possible to assume the appearance of fundamentally new properties in these complexes, for example, the magnetic or catalytic ones, due to interaction of the metal ions with each other or due to their synergistic effect. A special area of application of heterometallic complexes developed later is their use as precursors for mixed metal oxides, which are promising magnetic and catalytic materials, under thermolysis. The preparation of complex-oxide systems using heterometallic coordination compounds, from which the required oxide is formed after the removal of the organic part of the molecule, is of significant interest, since the composition of the expected oxide and some of its properties can be programmed already at the level of the molecular precursor. The usual time-consuming and laborious classic solid-phase synthesis of heterometallic oxides is often inefficient, especially in case of preparation of thin films or nanocrystals. The preparation of complex oxides by chemical homogenization, through a precursor in which metal atoms are included in one molecule, makes it possible to remove diffusion problems (inevitable in the case of classical solid-state synthesis), which not only accelerates the process, but also reduces the temperature of the reaction [7,8,9]. 

Compounds containing a transition metal as a part of an organometallic fragment represent a special and relatively poorly studied type among the multitude of 3d-3d′-heterometallic complexes. Such complexes are of both theoretical and practical interest, since they are potentially capable of combining the properties of a transition metal ion and an organometallic fragment; in addition, they also can serve as precursors for mixed oxide phases, which are valuable functional materials. For a long time, mainly ferrocene derivatives were studied [10,11], while the known derivatives of the other stable organometallic π-complexes, namely, cymantrene ((η^5^-C_5_H_5_)Mn(CO)_3_) [12,13,14,15], benchrotrene ((η^6^-C_6_H_6_)Cr(CO)_3_) [16,17,18], and cobaltocenium ([(η^5^-C_5_H_4_)_2_Co]^+^) [19,20] are still relatively few. We have shown previously that the cymantrenecarboxylate ligand CymCO_2_^−^ represents a convenient building block for construction of 3d-4f-heterometallic complexes having various structures [21,22]. In addition to purely rare-earth cymantrenecarboxylates, we have also obtained mixed 3d-4f-complexes containing, along with the lanthanide ions, the Mn^2+^ ions which appeared as a result of oxidative destruction of the cymantrene fragment during the synthesis [23,24]. In addition, we have obtained a mononuclear cymantrenecarboxylate complex of cadmium [Cd(κ^2^-CymCO_2_)(κ^1^-CymCO_2_)(phen)_2_]·2MeCN, which was found to undergo unusual single-crystal-to-single-crystal transformation [25], as well as a number of several cadmium cymantrenecarboxylates with mononuclear, binuclear and polymeric structures [26]. It was of interest to develop the chemistry of cymantrenecarboxylates further into the area of 3d-transition metals, in particular, to study the effect of a sterically complicated cymantrene fragment on the structural features of the complexes, as well as to explore their oxidative thermolysis, which can bring about bimetallic mixed oxides promising as functional materials. This paper describes the synthesis and the properties of new mononuclear cymantrenecarboxylate complexes of several 3d-transition metals having +2 oxidation state.

## 2. Results and Discussion

### 2.1. Synthesis and Structure of ***1***–***8***

By means of exchange reactions between transition metal salts and potassium cymantrenecarboxylate in various media in the presence of the appropriate ligands, a series of eight novel transition metals cymantrenecarboxylates was obtained. The complexes were characterized by single-crystal X-ray analysis; the selected bond lengths and angles for them are given in Table S1. All the compounds include mononuclear building blocks which exhibit significant structural diversity. In particular, in aqueous-organic media under conditions of slow evaporation of the organic phase, the transition metals (Co, Ni, Zn) form isostructural compounds **1**–**3** consisting of the isolated ions. Interestingly, the similar process with use of the copper chloride led to the previously described binuclear complex [Cu_2_(CymCO_2_)_4_(THF)_2_] [12], while the lanthanides mostly formed coordination polymers under similar conditions [27,28].

**Figure 1:**
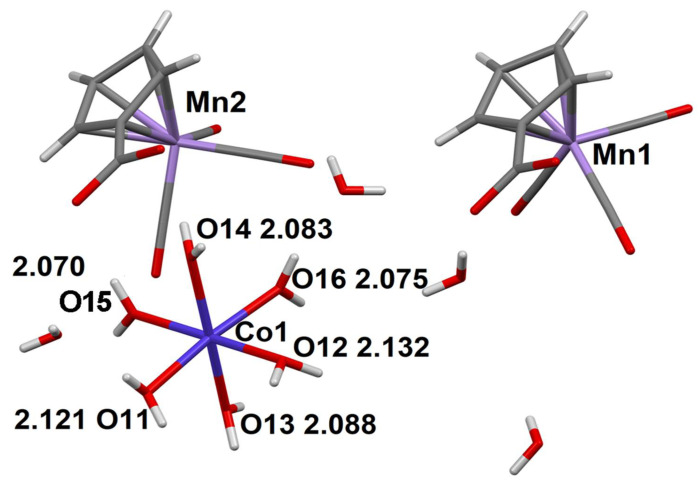
Asymmetric unit in the structure of compound **1**. The distances between the coordinating oxygen atoms of aqua ligands and the Co^2+^ ion are given (Å).

The structures of **1**–**3** will be considered, taking compound **1** as an example. The structure of **1** is formed of octahedral cationic [Co(H_2_O)_6_]^2+^ complexes, cymantrenecarboxylate CymCO_2_^−^ anions and lattice water molecules (Figure 1). The branched system of hydrogen bonds (H-bonds; Table S2) brings about formation of a layered structure.

**Figure 2:**
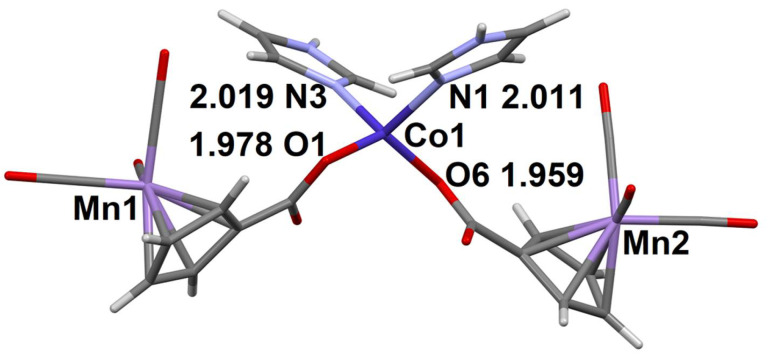
The structure of complex molecule in compound **4**. The distances between the coordinating atoms of the ligands and the Co^2+^ ion are given (Å).

The coordination surrounding of the Co atom in the structure of compound **4** (Figure 2) is a highly distorted tetrahedron (bond angles around the Co atom are in the range of 97.4–129.8°, see Table S1) formed by carboxylate O atoms and N atoms of imidazole. Two N-H…O H-bonds (Table S2) formed with the participation of non-coordinating carboxyl atoms of CymCO_2_^−^ ligands combine the complex molecules into layers.

**Figure 3:**
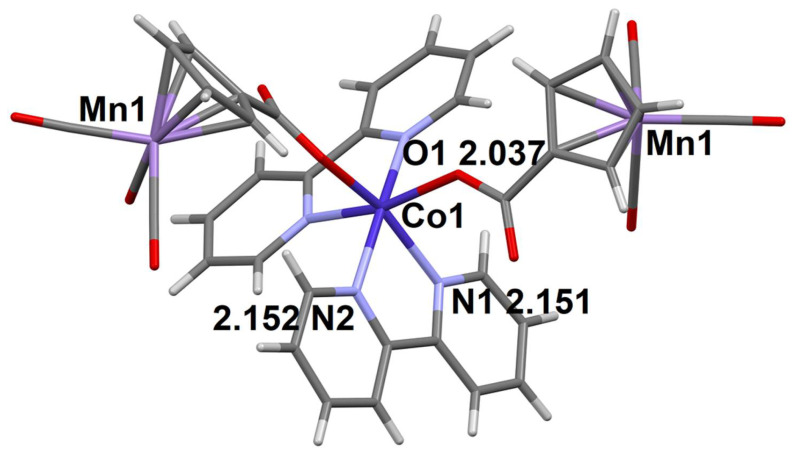
The structure of complex molecule in **5**. The distances between the coordinating atoms of the ligands and the Co^2+^ ion are given (Å).

The complex molecule [Co(CymCO_2_)_2_(bpy)_2_] in the structure of compound **5** is located on a twofold axis, the coordination number of the Co atom is 6, and the coordination polyhedron is a distorted octahedron. Two oxygen atoms of the coordinated CymCO_2_^−^ anions are in cis-position relatively to each other (Figure 3). The CymCO_2_^−^ group is turned in such a way that the O…X distance (where O is the non-coordinating carboxyl atom, and X is the center of bpy molecule) is 3.20 Å.

**Figure 4:**
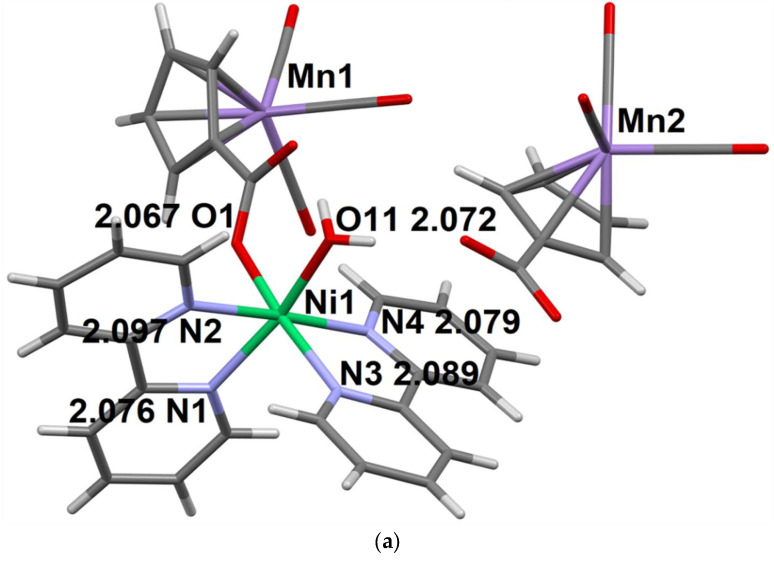

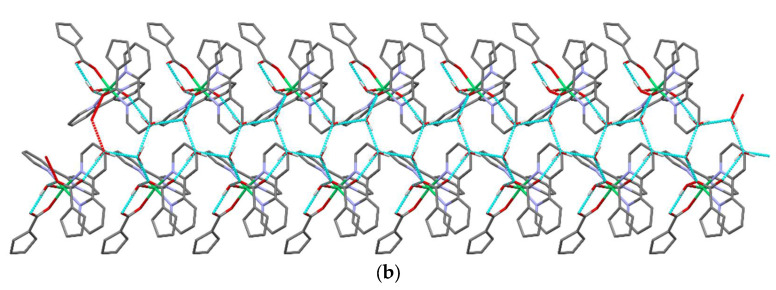
The molecular structural unit of compound **6** (**a**) and double 1D-chains in its crystal structure (**b**). The distances between the coordinating atoms of the ligands and the Ni^2+^ ion are given (Å).

In contrast to the structure of complex **5**, in the Ni derivative with bpy ligand **6**, the coordination environment of the 3d metal (Ni) is formed of two bpy molecules, the oxygen atoms of the monodentally coordinated CymCO_2_^−^ anion and H_2_O molecule; the oxygen atoms are in cis-positions relatively to each other (Figure 4). The second CymCO_2_^−^ anion is not coordinated to the metal ion. The structure of compound **6** also contains MePh and H_2_O lattice molecules. Six O-H…O H-bonds (Table S2) combine structural units (with the exception of MePh molecules) into double 1D-chains (Figure 4b).

**Figure 5:**
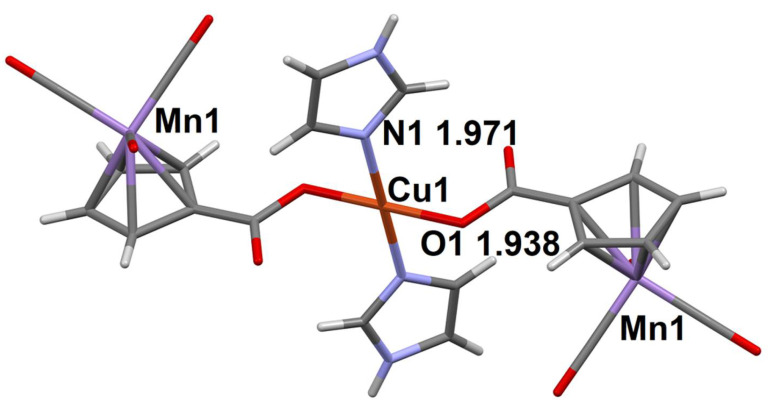
The structure of complex molecule in **7**. The distances between the coordinating atoms of the ligands and the Cu^2+^ ion are given (Å).

The Cu atom in the structure of complex molecule in compound **7** (Figure 5) is located at the center of inversion, its square surrounding is formed of the nitrogen atoms of two imidazole molecules and two oxygen atoms of CymCO_2_^−^ ligands. The N-H…O H-bonds (O is the non-coordinating carboxyl atom of CymCO_2_^−^ ligand, see Table S2) bind the molecular complexes into layers. 

**Figure 6:**
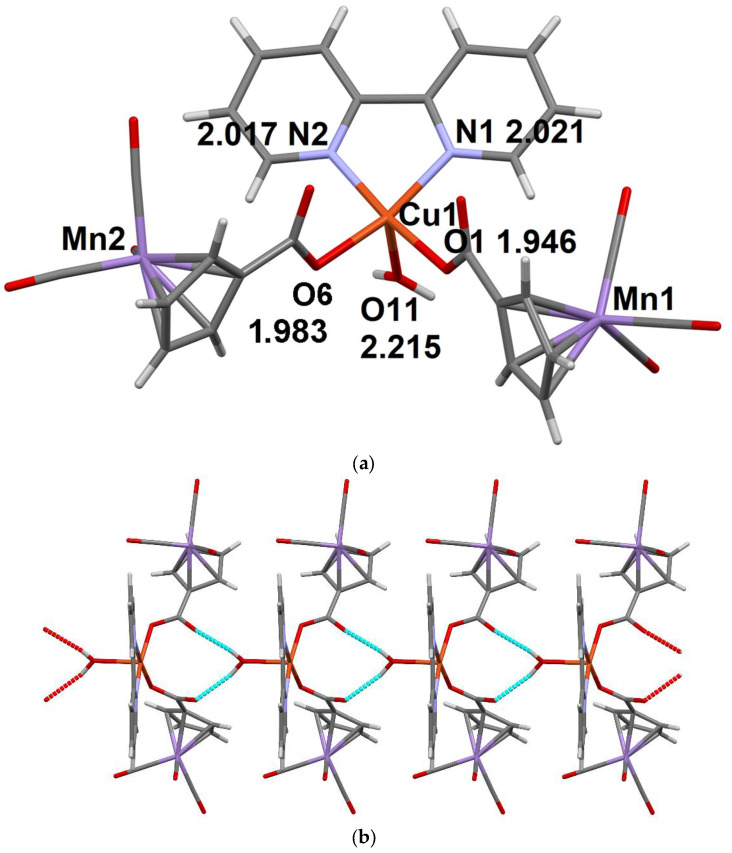
The molecular structural unit of compound **8** (**a**) and 1D-chain in its crystal structure (**b**). The distances between the coordinating atoms of the ligands and the Cu^2+^ ion are given (Å).

The coordination number of the Cu atom in compound **8** is 5, the coordination polyhedron is a square pyramid, the apical position of which is occupied by the coordinated H_2_O molecule (Figure 6a). Two O-H...O H-bonds (Table S2) are involved in the formation of the R_2_^2^(10) cycle, and combine the complex molecules into a 1D-chain (Figure 6b).

Some of the previously known transition metal cymantrenecarboxylates had bi- and polynuclear structures typical of transition metal carboxylates, in particular, the Cu, Co and Ni complexes had binuclear paddle-wheel structure [12,13]; for Co, the trinuclear complexes were also obtained [13]. At the same time, it is known that the steric complication of the ligand facilitates formation of mononuclear complexes [29], therefore, the bulkiness of the CymCO_2_^−^ ligand can explain the formation of mononuclear carboxylates in all of the cases under consideration.

To date, CCDC database (version 5.42, September 2021) [30] contains data on 19 mononuclear cymantrenecarboxylates of d-metals (Table S3). In 18 of them, CymCO_2_^−^ is coordinated by the 3d-metal atom, and only in the complex [Ni(phen)_3_](CymCO_2_)_2_·4H_2_O (phen = 1,10-phenanthroline) [31] there are “free” non-coordinated CymCO_2_^−^ anions. In all the octahedral (or pseudo-octahedral) complexes with bidentate auxiliary ligands (bpy, phen), CymCO_2_^−^ ligands are in the cis-position to each other, while in the complexes with monodentate ligands (MeOH, 2,6-Me_2_Py, pyrazole) they are in the trans-positions. In the new compounds considered in this article, the same patterns of molecular structure are observed. In compound **6**, the presence of both the coordinated and the free CymCO_2_^−^ anions was found for the first time.

### 2.2. Magnetic Properties of Co Complexes ***1***, ***4***, and ***5***

Among the complexes obtained, the most interesting in terms of magnetic properties are the cobalt compounds, since a large number of single-ion magnets (SIMs) is known among the mononuclear Co^2+^ complexes [32,33,34]. SIMs represent a subtype of single-molecule magnets, SMMs, which are the substances whose molecules below a certain “blocking” temperature exhibit the properties of individual magnets. Such compounds are of interest as a possible means for ultra-dense storage of information, since they potentially can store one bit of information in each molecule [35,36,37]. Complexes containing Co(II) are the most prominent among the 3d-element compounds as potential SMMs. In particular, for the cobalt complexes, the record SMM characteristics among the transition metal complexes were obtained [38,39,40]. In this regard, magnetic studies of complexes **1**, **4**, and **5** were carried out in constant and alternating magnetic fields. 

**Figure 7:**
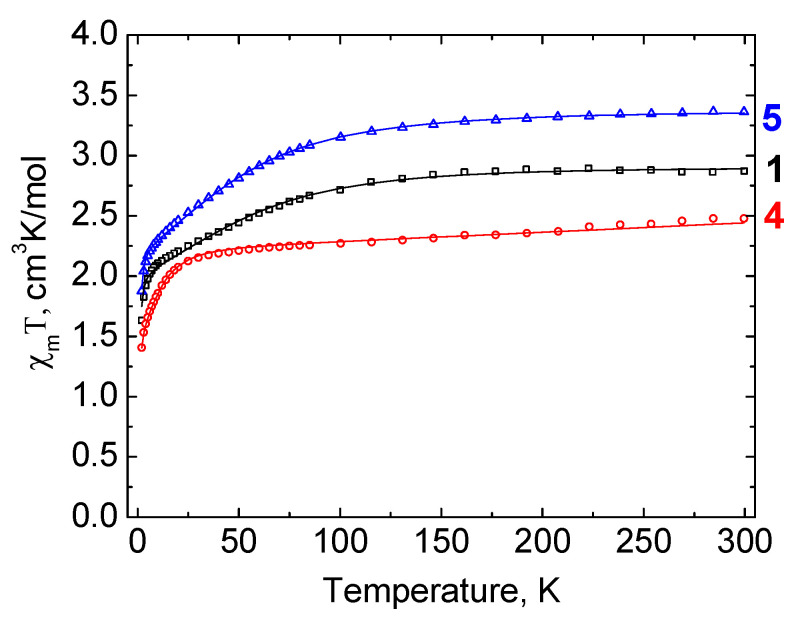
Temperature dependence of χ_m_T for complexes **1**, **4**, and **5** under magnetic field of 5000 Oe. Solid lines represent the approximation by PHI software [42].

The temperature dependences of χ_m_T for the studied compounds are shown in Figure 7. It should be noted that the Mn(I) atoms present in the cymantrenyl fragments of **1**, **4**, and **5** are diamagnetic (low-spin d^6^ configuration) and do not contribute to the magnetism of the complexes. For complex **1**, the value of χ_m_T at 300 K is 2.87 cm^3^mol^−1^K, which corresponds to the magnetic moment (μ_eff_) value of 4.79 μ_B_. This value is larger than the spin-only value for S = 3/2 system (3.87 μ_B_), but is almost equal to 4.8 μ_B_, which is a usual experimental value for a Co^2+^ ion having 3d^7^ electron configuration with non-zero orbital contribution to the magnetic moment [41]. With the temperature lowering, χ_m_T decreases, smoothly down to ≈100 K, and then more abruptly, changing to a drop below 10 K. At the temperature of 2 K, the value of χ_m_T reaches 1.63 cm^3^mol^−1^K (μ_eff_ = 3.63 μ_B_). 

For complex **4**, the value of χ_m_T at 300 K is 2.48 cm^3^mol^−1^K, which corresponds to the magnetic moment μ_eff_ of 4.45 μ_B_. This value is also larger than the spin-only value which indicates a significant orbital contribution. With a decrease in temperature, χ_m_T descends smoothly down to 40 K, and then sharply. At a temperature of 2 K, χ_m_T is equal to 1.40 cm^3^mol^−1^K (μ_eff_ = 3.35 μ_B_). 

For complex **5**, the χ_m_T value at 300 K is 3.36 cm^3^mol^−1^K, which corresponds to the magnetic moment μ_eff_ = 5.19 μ_B_. This value is close to the highest of typical values for the Co^2+^ octahedral complexes with a large orbital contribution to the magnetic moment (5.2 μ_B_ [43]). The decrease in temperature is accompanied by a gradual descent in χ_m_T value, which becomes sharper below 100 K. At the temperature of 2 K, χ_m_T reaches the value of 1.87 cm^3^mol^−1^K (μ_eff_ = 3.87 μ_B_). In all of the cases, the decrease in χ_m_T can be due to the thermal depopulation of the high energy Kramers doublets of the Co^2+^ ion or to the zero-field splitting. The temperature dependences of χ_m_T were approximated by the PHI software [42] using the following effective spin Hamiltonian:

$$Ĥ=D{Ŝ}_{z}{}^{2}+\mu_{B}(g_{x}{Ŝ}_{x}+g_{y}{Ŝ}_{y}+g_{z}{Ŝ}_{z})B$$

The best-fit values are presented in Table 1. The contributions of zero-field splitting and Zeeman effect were taken into account. Regarding complex **4**, the temperature-independent paramagnetism parameter (TIP) was also applied for calculation improvement.

As it can be seen from Figures S1–S3, the out-of-phase component of magnetic susceptibility χ″, which indicates the presence of slow magnetic relaxation, appears only under non-zero applied field for all three complexes, **1**, **4**, and **5**. This effect originates from the suppression of quantum tunneling by the outer field [22]. Therefore, we had to study the dynamic magnetic behavior of compounds **1**, **4**, and **5** in magnetic fields up to 5000 Oe to confirm the existence of slow relaxation of magnetization in them and to evaluate its parameters. Isotherms of in-phase χ′(ν) and out-of-phase χ″(ν) dependences for complexes **1**, **4**, and **5** taken under experimentally found optimal H_dc_ fields (2500 Oe for **1**, 1500 Oe for **4**, and 1000 Oe for **5**) are shown in Figures S4–S6, respectively. 

**Figure 8:**
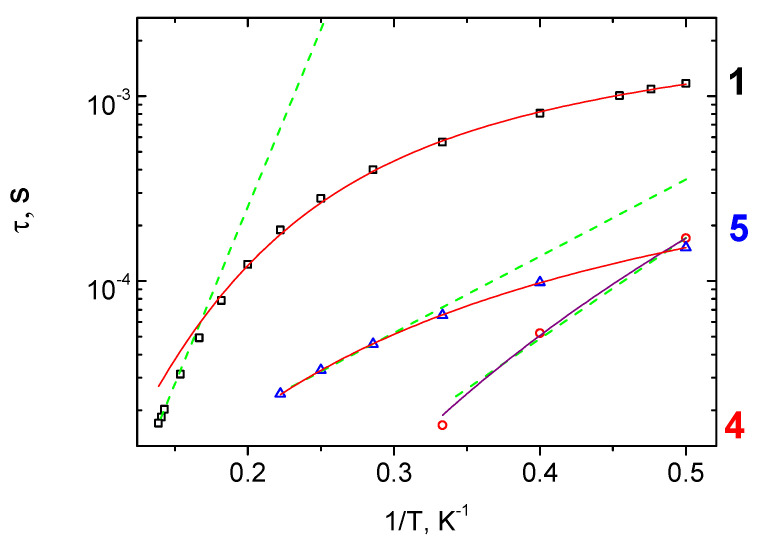
τ vs. 1/T plots for **1**, **4** and **5** under optimal dc-fields. Dashed lines represent approximations of high-temperature range by equation corresponding to Orbach relaxation mechanism [32,33,34]. Solid lines represent the fittings using Raman (**4**) and combined contribution of Raman and direct (**1**, **5**) relaxation mechanisms.

Approximations of these dependences by the generalized Debye model [44] allowed us to create temperature dependences of the relaxation time, τ(1/T), presented in Figure 8. Three independent mechanisms are known to dominate the magnetic relaxation process in case the quantum tunneling is suppressed, namely, Raman, Orbach-like (thermally activated), and direct relaxation pathways, which are summarised in the following equations: τ^−1^ = C_Raman_T^nRaman^, τ^−1^ = τ_0_^−1^exp(-Δ_eff_/k_B_T), and τ^−1^ = AT^4^, respectively [22]. The best correspondence to the experimental data for τ(1/T) approximation was achieved by use of Raman relaxation mechanism for **4,** and of the sum of Raman and the direct relaxation mechanisms for **1** and **5**. Relaxation parameters and SMM characteristics obtained are presented in Table 2.

### 2.3. Solid-State Thermolysis of Complexes **1**–**8** in Air Atmosphere

Previously, we have shown that thermolysis of lanthanide cymantrenecarboxylate complexes brings about multiferroic rare-earth manganites LnMnO_3_ or LnMn_2_O_5_ as the solid products, and the type of the manganite formed is determined by the ratio of metals in the initial complex [21,28,45]. The complexes considered in this article have a ratio of M:Mn = 1:2; consequently, it could be assumed that as a result of their thermolysis in air, spinel-type manganites MMn_2_O_4_ would be formed. Transition metal manganites of spinel type are valuable functional materials. In particular, they are of interest as magnetic materials [46,47,48], catalysts [49,50], photovoltaic materials [51], electrode materials [52,53], and materials for negative temperature coefficient thermistors [54]. Therefore, we carried out the solid-state thermolysis of the obtained compounds **1**–**8** in the air flow in the temperature range of 25–950 °C.

**Figure 9:**
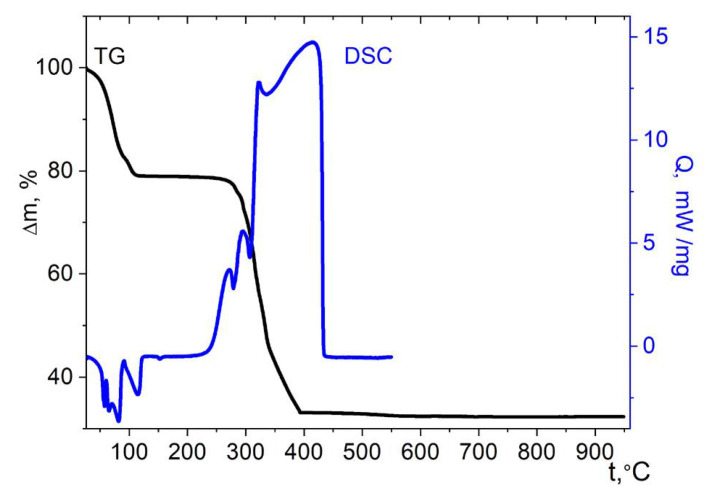
Thermolysis of complex **1** under air.

The weight loss in the case of the cobalt complex with aqua ligands **1** already begins at 25 °C. Three stages can be distinguished on the TG curve (Figure 9). The first stage ends at about 120 °C, with a weight loss of ≈21.5%. This weight loss is close to the mass of nine water molecules of ten present in the molecular unit (22% theor.). Complex effects are noted on the DSC curve in this range, which indicate the breaking of the H-bonds and a significant rearrangement of the structure. Then, up to a temperature of 260 °C, a plateau is noted corresponding to the region of stability of the dehydrated phase. At higher temperature, the oxidation of the cymantrenecarboxylate fragment accompanied by a sharp mass loss begins. A pronounced exotherm is noted on the DSC curve above this temperature, which ends at 440 °C. The final product of thermolysis of complex **1** at 950 °C, according to XRD data, is the tetragonal spinel CoMn_2_O_4_ (Figure S7). The total weight loss (67 ± 2%) is also in good agreement with the expected CoMn_2_O_4_ weight in case it is formed as the only solid product (31.8% of the initial mass).

**Figure 10:**
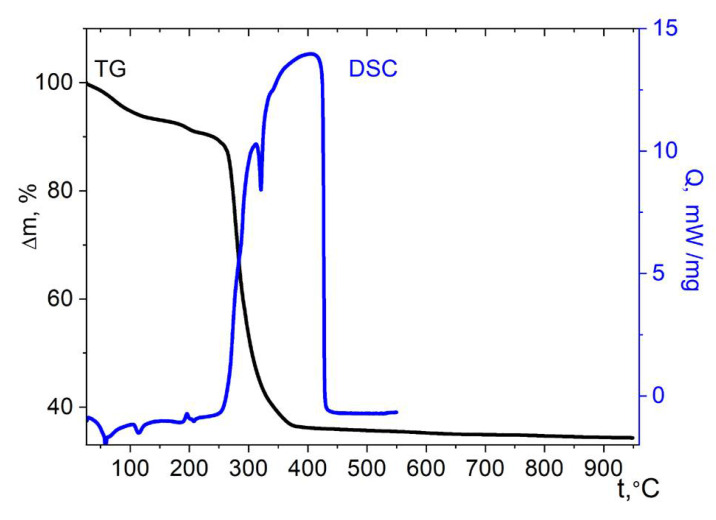
Thermolysis of complex **2** under air.

Thermolysis of the hydrated nickel compound **2** has significant differences from the cobalt analogue (Figure 10). Two stages can be distinguished on the TG curve. At the first stage (25–260 °C), the weight loss begins almost at the start of the measurement and is equal to ≈11%. This weight loss is in good agreement with the content of four guest water molecules (9.8%); the ease of their removal also confirms their non-coordinated nature. Moderate effects are present in the DSC curve. Above 260 °C, a sharp weight loss begins, which is associated with the onset of oxidation of the cymantrenecarboxylate fragment. In the DSC experiment conditions, a strong exotherm starts above this temperature. The exothermic process is superimposed with the endothermic process of removing water molecules coordinating Ni^2+^ ions, which occurs in approximately the same temperature range [55]. The superposition of the endothermic effect of water removal can explain the splitting of the exotherm on the DSC curve. The final product of thermolysis is the cubic spinel NiMn_2_O_4_ (Figure S8). The mass of the residue (34.3 ± 2%) is close to the expected mass of the NiMn_2_O_4_ mixed oxide (31.7% of the initial mass).

**Figure 11:**
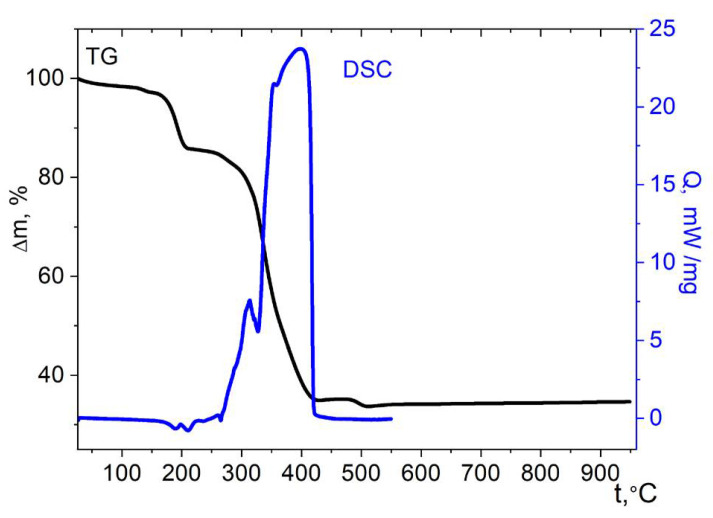
Thermolysis of complex **3** under air.

The thermal behavior of zinc complex **3** indicates its somewhat higher stability compared to compounds **1** and **2** (Figure 11). Up to a temperature of 172 °C, a loss of 4.8% of the mass occurs, which corresponds to the removal of two molecules of lattice water (4.86% theor.). Further, up to a temperature of 205 °C, a sharp loss of 10% of the mass occurs, which corresponds to the loss of four H_2_O molecules. At the same time, insignificant endothermic effects are observed on the DSC curve. Then, up to the temperature of 260 °C, a region of stability of the partially dehydrated product is observed. Active oxidation of the organic part also begins at slightly higher temperature than in cases of **1** and **2**, namely, at ≈280 °C. The final product of thermolysis is the tetragonal spinel ZnMn_2_O_4_ (Figure S7). The mass of the residue (34.1 ± 2%) at the end of the process is close to the theoretically expected for ZnMn_2_O_4_ as the only solid product (32.3%).

**Figure 12:**
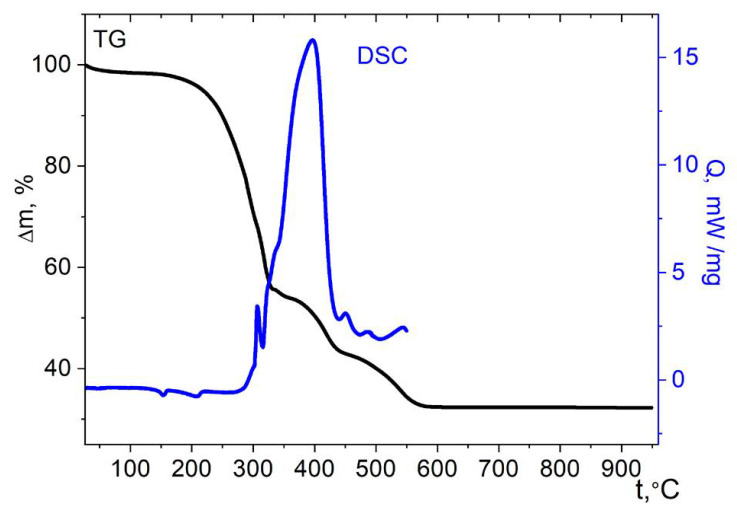
Thermolysis of complex **4** under air.

The complex of Co with the imidazole ligand **4** is thermally stable up to ≈200 °C, the mass loss below this temperature is insignificant (Figure 12). The minor thermal effects below this temperature, most likely, correspond to the breaking of the H-bonds. The pronounced mass loss starts above the specified temperature. It should be noted that the weight loss during thermolysis of a nickel complex with imidazole ligands [Ni(imz)_6_](NO_3_)_2_ starts approximately in the same temperature range [56]. The oxidation of complex **4** under the conditions of the DSC experiment begins above 292 °C; it is accompanied by a complex exotherm. Thermolysis of compound **4** is complicated, which indicates the simultaneous occurrence of several processes. The weight loss is completed at 575 °C, which is significantly higher than for the cases of the other cobalt cymantrenecarboxylate complexes under consideration, and the thermal effects are observed over the entire temperature range studied by the DSC method. Most likely, this is due to the formation of an intermediate solid products of imidazole thermolysis. The final product of thermolysis is the tetragonal spinel CoMn_2_O_4_ (Figure S7); the mass of the remainder is 32.3 ± 2% of the starting value, which is close to the theoretically expected one (33.8%).

**Figure 13:**
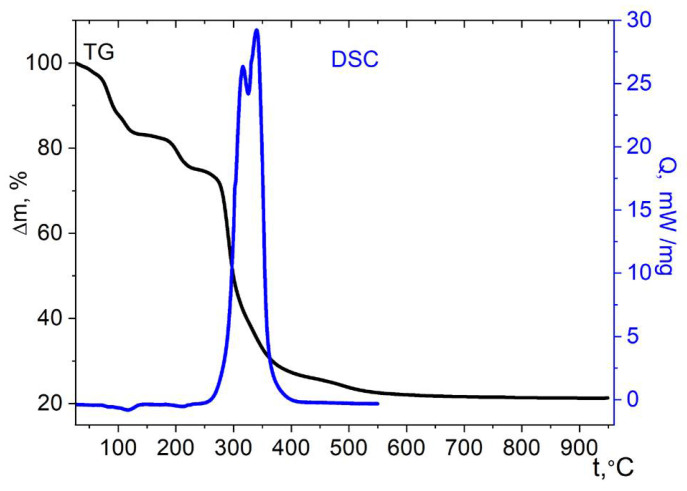
Thermolysis of complex **5** under air.

Thermolysis of compound **5** begins with the removal of the lattice toluene molecules, which corresponds to a loss of ≈16.8% of the mass in the range of 25–130 °C (the calculated content of toluene is 17.4%). Further, up to a temperature of about 200 °C, a region of relative stability of the desolvated complex is noted (Figure 13). Weight loss at a higher temperature, before the oxidation starts, can be explained by the partial removal of the volatile bipyridyl ligand. At ≈260 °C, an active exothermic oxidation of the organic part of the complex begins, leading, according to powder X-ray data, to the tetragonal spinel CoMn_2_O_4_ (Figure S7). The mass of the solid residue (21.4 ± 2%) corresponds well to the calculated one (22.2%).

**Figure 14:**
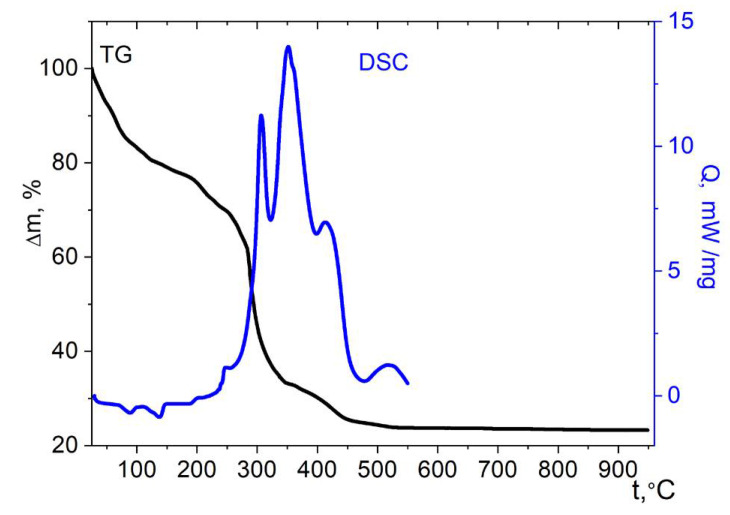
Thermolysis of complex **6** under air.

The mass loss during the thermolysis of complex **6**, according to TG data, begins at 25 °C already, and the mass loss corresponding to the elimination of water and toluene lattice molecules (8.5%) is achieved at 50 °C (Figure 14). The further intense weight loss is possibly associated with the removal of a water molecule and one of the bpy ligands from the inner sphere, with the introduction of the outer cymantrenecarboxylate ligand into the inner sphere of the complex. However, the corresponding weight loss (down to 73.64% of the initial) is achieved at a temperature of 215 °C, when the coordinated bpy is not firmly withheld and its further removal is possible, as it was found for nickel chloride [57] and bromide [58] complexes with bpy ligand. The weight loss corresponding to the complete elimination of neutral ligands (57.3% of the initial mass at 287 °C) falls into the region of active oxidation of organic matter, which starts at ≈250 °C; therefore, it is not possible to separate the stages of this process. The final product of thermolysis, according to powder XRD data, is the cubic spinel NiMn_2_O_4_ (Figure S8). The mass of the solid residue (23.3 ± 2%) corresponds to the expected one (24% of the initial value).

**Figure 15:**
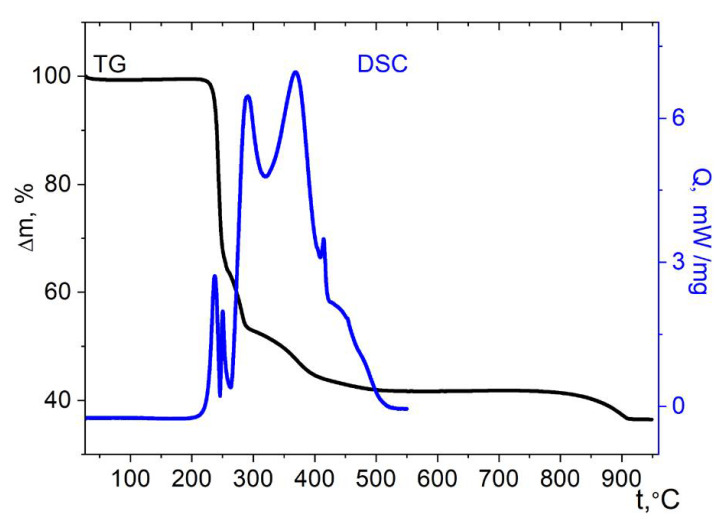
Thermolysis of complex **7** under air.

Similarly to the cobalt complex with imidazole **4**, the copper compound with the same ligand **7** is relatively thermally stable (Figure 15). The weight loss begins at 235 °C, which is probably due to the onset of decomposition of the cymantrene fragment. The start of the weight loss coincides with the complex effect on the DSC curve, which is, most likely, associated with the intramolecular reduction of Cu^2+^, which is a relatively strong oxidizer, by the organometallic part of the molecule. The complexity of the effect is probably determined by the simultaneously occurring processes, i.e., the exothermic reduction of copper and the endothermic removal of volatile fragments of the molecule. This effect, at a temperature of 265 °C changes to an intense exotherm, indicating the oxidation of organic matter by the air oxygen. Up to 500 °C, 58% of the initial mass is lost; above 800 °C, the mass loss takes place again, which is probably associated with the burnout or decomposition of the stable intermediate; the total loss at 950 °C is 63.5 ± 2%. The mass of the residue (36.5 ± 2%) is close to the calculated one for CuMn_2_O_4_ gross formula (34.2%). According to XRD data, the thermolysis product contains the mixture of CuO and the cubic CuMn_2_O_4_ phase (Figure S9).

**Figure 16:**
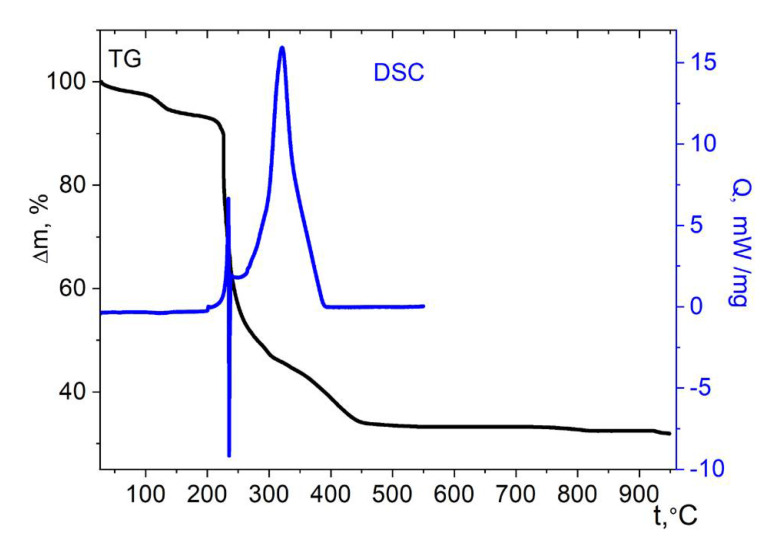
Thermolysis of complexes **8** under air.

Thermolysis of the cymantrenecarboxylate complex of copper with bipyridyl ligand **8** begins with the removal of the coordinated water (Figure 16). The mass loss corresponding to the water content (2.46%) is observed at 100 °C; the further small mass loss (≈4%) before the onset of redox processes at 220 °C is probably associated with the removal of a part of the volatile bipyridyl ligand. Similarly to the case of another copper-containing compound **7**, there is an ambiguous effect on the DSC curve associated with the onset of weight loss and related, probably, with Cu^2+^ reduction, which is superimposed with the removal of the volatile part of the molecule (for example, bpy or CO ligands). Under the conditions of the TG experiment, the redox processes are completed at the temperature of ≈450 °C; no significant further changes in mass are observed. The mass of the solid residue (32.0 ± 2%) is close to the calculated one for CuMn_2_O_4_ (32.4%). The product of thermolysis, according to powder XRD data, contains CuO and the cubic spinel CuMn_2_O_4_ (Figure S9).

## 3. Materials and Methods

The following commercial reagents and solvents were used for the syntheses: hydrated metal chlorides MCl_2_·*n*H_2_O and ZnSO_4_·6H_2_O from Alfa Aesar, cymantrene, 2,2′-bipyridyl and imidazole from Aldrich, and solvents (MeOH, THF, DMSO, C_6_H_5_Me, hexane) from Alfa Aesar. Carboxycymantrene CymCO_2_H was synthesized according to a known procedure [59]. Before use in the synthesis, CymCO_2_H was sublimed in vacuo to remove the traces of Mn^2+^. All experiments with the solutions of the compounds were carried out in foil-wrapped vessels to prevent photolysis. Methanol was distilled over magnesium before use; THF was distilled over LiAlH_4_; toluene was successively distilled over P_2_O_5_ and sodium. 

Elemental analysis was carried out using an EA1108 85 automatic C,H,N,S analyzer (Carlo Erba Instruments). The attenuated total reflection infrared (ATR-IR) spectra were recorded in the range of 600–1600 cm^−1^ on a Bruker ALPHA instrument. 

The magnetic susceptibility measurements of cobalt complexes **1**, **4**, and **5** were performed on a Quantum Design susceptometer PPMS-9. The dc measurements were performed under an external magnetic field of 5000 Oe in the temperature range 2–300 K. The temperature-dependent effective magnetic moment was calculated by the equation μ_eff_ = *[(3k/Nβ^2^*)·*χT]^1/2^* *≈ (8χT)^1/2^*, where *N* is Avogadro’s number, *k* is the Boltzmann constant, and *β* is the Bohr magneton. For ac-magnetic susceptibility measurements of all the samples, oscillating ac fields of 0–5000 Oe for complex **1**, and 0–2500 Oe for complexes **4** and **5**, within frequency ranges 0–10,000 Hz, respectively, were applied. These settings allowed one both to avoid sample heating at low temperatures (which may occur when modulation amplitudes and frequency are high) and to obtain the best signal-to-noise ratio. Measurements were performed on grinded polycrystalline samples sealed in a polyethylene bag and covered with mineral oil in order to prevent field-induced orientation of the crystals. The paramagnetic components of the magnetic susceptibility *χ* were determined taking into account the diamagnetic contribution evaluated from Pascal constants as well as the contributions of the sample holder and mineral oil.

### 3.1. Synthesis of [M(H_2_O)_6_](CymCO_2_)_2_·4H_2_O, (M = Co, Ni, Zn; ***1***–***3***)

Potassium hydroxide (56 mg, 1 mmol) and CymCO_2_H (248 mg, 1 mmol) were dissolved in methanol (5 mL). The solution was stirred for 20 min at room temperature. Then, a solution of CuCl_2_·2H_2_O, CoCl_2_·6H_2_O or ZnSO_4_·6H_2_O (0.5 mmol) in water (5 mL) was added with vigorous stirring, and THF (6 mL) was added to the reaction mixture. The mixture was refluxed in a water bath for 10 min. After cooling, the mixture was filtered through a glass filter and left to evaporate slowly at room temperature in a flask wrapped up with an aluminum foil, to avoid photolysis of the cymantrene fragment. The neck of the flask was plugged with a cotton wool to make evaporation more equable. Under these conditions, the products were crystallized within ten days. Zinc derivative contained an admixture of CymCO_2_H from which it was washed with chloroform. The yields of compounds **1**–**3** were 55–65%.

Complex **1**. Calculated for C_18_H_28_CoMn_2_O_20_: C, 29.49; H, 3.85. Found: C, 29.55; H, 3.80. FTIR of **1**: 3616 w, 3114w, 2018 m, 1906 vs, 1674 w, 1566 m, 1473 s, 1388 s, 1350 s, 1215 m, 1198 m, 1188 m, 1059 w, 1031 m, 923 w, 850 m, 838 m, 777 m, 665 m, 626 vs, 533 vs, 484 s, 462 s.

Complex **2**. Calculated for C_18_H_28_NiMn_2_O_20_: C, 29.50; H, 3.85. Found: C, 29.57; H, 3.80. FTIR of 2: 3622 w, 3117w, 2017 m, 1906 vs, 1678 w, 1573 m, 1473 s, 1387 s, 1358 s, 1215 m, 1195 m, 1184 m, 1059 w, 1030 m, 922 w, 850 m, 841 m, 780 m, 664 m, 625 vs, 531 vs, 492 s, 456 s.

Complex **3**. Calculated for C_18_H_28_ZnMn_2_O_20_: C, 29.23; H, 3.82. Found: C, 29.25; H, 3.79. FTIR of 3: 3584 w, 3120w, 2018 m, 1908 vs, 1688 w, 1568 m, 1476 s, 1382 s, 1357 s, 1214 m, 1198 m, 1063 w, 1031 m, 922 w, 850 m, 841 m, 789 s, 666 m, 630 vs, 532 s, 496 m, 449 m.

### 3.2. Synthesis of [Co(CymCO_2_)_2_(imz)_2_] (***4***), [Co(CymCO_2_)_2_(bpy)_2_]·2PhMe (***5***), [Ni(CymCO_2_)(bpy)_2_(H_2_O)][CymCO_2_]·0.5MePh·2H_2_O (***6***), and [Cu(CymCO_2_)_2_(bpy)(H_2_O)] (***8***)

Potassium hydroxide (56 mg, 1 mmol) and CymCO_2_H (248 mg, 1 mmol) were dissolved in methanol (5 mL). The solution was stirred for 20 min at room temperature. A solution of CoCl_2_·6H_2_O (119 mg; 0.5 mmol), or NiCl_2_·6H_2_O (119 mg; 0.5 mmol), or CuCl_2_·2H_2_O (67 mg; 0.5 mmol) and imidazole (68 mg; 1 mmol) or 2,2′-bipyridyl (156 mg; 1 mmol) in 5 mL of methanol was added. The mixture was stirred for two hours and evaporated to dryness. The residue was redissolved in CH_3_CN (5 mL) or in CH_3_OH (5 mL) in case of 8, then 9 mL of toluene was added and the mixture was refluxed for 10 min. Then, the solution was filtered and left to evaporate slowly. The products crystallized during a two-week period. The yields were 144 mg (42%) for **4**, 335 mg (64%) for **5**, 275 mg (57%) for **6** and 223 mg (61%) for **8**.

Complex **4**. Calculated for C_24_H_16_CoMn_2_N_4_O_10_: C, 41.82; H, 2.34; N, 8.13. Found: C, 41.80; H, 2.30; N, 8.15. FTIR of **4**: 3137 m, 3062 w, 2960 w, 2864 w, 2015 m, 1954 m, 1916 s, 1705 w, 1582 m, 1538 m, 1479 m, 1416 m, 1382 s, 1352 s, 1327 m, 1259 m, 1239 m, 1218 w, 1197 m, 1181 m, 1139 m, 1093 m, 1066 s, 1028 m, 950 m, 925 m, 847 m, 784 s, 745 s, 658 s, 628 vs, 579 m, 529 s, 490 m, 472 m.

Complex **5**. Calculated for C_52_H_40_CoMn_2_N_4_O_10_: C, 59.50; H, 3.84; N, 5.34. Found: C, 59.45; H, 3.80; N, 5.40. FTIR of **5**: 3107 w, 3026 w, 2952 w, 2923 w, 2854 w, 2012 m, 1906 s, 1599 m, 1537 m, 1491 m, 1472 s, 1442 m, 1415 m, 1388 s, 1350 s, 1313 m, 1249 m, 1239 m, 1210 w, 1191 m, 1176 m, 1156 m, 1119 m, 1103 m, 1039 s, 1023 m, 976 m, 917 m, 813 m, 799 m, 769 s, 733 s, 695 m, 664 s, 625 vs, 563 m, 538 s, 488 m, 463 s.

Complex **6**. Calculated for C_41.50_H_34_Mn_2_N_4_NiO_13_: C, 51.64; H, 3.55; N, 5.80. Found: C, 51.59; H, 3.50; N, 5.84. FTIR of **6**: 3304 w, 3109 w, 3028 w, 2013 m, 1909 s, 1599 m, 1565 m, 1531 m, 1494 m, 1473 m, 1443 m, 1415 m, 1391 s, 1354 s, 1314 m, 1249 m, 1212 w, 1192 m, 1174 w, 1157 m, 1118 w, 1105 w, 1027 m, 976 w, 916 w, 851 w, 817 m, 800 m, 770 m, 735 m, 698 w, 665 s, 628 s, 566 m, 540 s, 490 m, 467 m.

Complex **8**. Calculated for C_28_H_18_CuMn_2_N_2_O_11_: C, 45.95; H, 2.48; N, 3.83. Found: C, 46.02; H, 2.50; N, 3.85. FTIR of 8: 3305 w, 3108 w, 2011 m, 1905 s, 1599 m, 1558 m, 1493 m, 1470 m, 1444 m, 1413 m, 1384 s, 1384 s, 1347 s, 1251 m, 1215 m, 1191 m, 1173 m, 1156 m, 1120 w, 1106 w, 1057 w, 1029 m, 1019 m, 979 w, 919 w, 849 w, 797 m, 769 m, 730 m, 695 w, 666 s, 629 vs, 579 m, 538 s, 490 m, 464 m.

### 3.3. Synthesis of [Cu(CymCO_2_)_2_(imz)_2_] (***7***)

Potassium hydroxide (56 mg, 1 mmol) and CymCO_2_H (248 mg, 1 mmol) were dissolved in methanol (5 mL). The solution was stirred for 20 min at room temperature. Then, a solution of CuCl_2_·2H_2_O (67 mg; 0.5 mmol) and imidazole (68 mg; 1 mmol) in 5 mL of methanol was added. The mixture was stirred for two hours and evaporated to dryness. The residue was dissolved in hot (~150℃) DMSO (8 mL), the solution was filtered through a glass filter and left to cool slowly on the oil bath. The product crystallized during a day. The liquid was decanted, the crystals were washed with toluene and hexane successively, and air-dried. The yield was 180 mg (52%).

Complex **7**. Calculated for C_24_H_16_CuMn_2_N_4_O_10_: C, 41.55; H, 2.32; N, 8.08. Found: C, 41.61; H, 2.33; N, 8.10. FTIR of 7: 3148 w, 3121 w, 3042 w, 2958 w, 2939 w, 2859 w, 2019 m, 1942 m, 1913 s, 1609 w, 1557 s, 1545 s, 1474 m, 1420 m, 1389 s, 1367 m, 1330 m, 1268 m, 1245 w, 1197 m, 1144 m, 1103 w, 1078 m, 1038 w, 955 w, 921 m, 902 m, 847 m, 798 m, 757 m, 667 m, 632 vs, 573 m, 544 m, 530 s, 491 m, 476 m.

### 3.4. X-ray Data Collection

Experimental data were collected on a Bruker SMART APEX2 instrument [60] (Table S4). Absorption was taken into account by a semiempirical method based on equivalents using SADABS [61]. The structures were determined using a combination of the direct method and Fourier syntheses. The positions of the H atoms were partially calculated from geometric considerations, partially localized from the difference Fourier syntheses. The lattice MePh molecules in structures **5** and **6** are disordered. All four tested needle-like “single crystals” **7** were intergrowths, which made it impossible to adequately take into account the absorption and determined a high R_int_ = 0.127. Attempts to take into account pseudomerohedra006C twinning were unsuccessful. All the calculations were carried out using SHELXS and SHELXL software [62].

Powder X-ray diffraction analysis of thermal decomposition products of **1**–**8** was carried out on a Bruker D8 ADVANCE X-ray Diffractometer (CuKα, Ni-filter, LYNXEYE detector, reflection geometry). 

X-ray diffraction studies were performed at the Centre of Shared Equipment of IGIC RAS.

### 3.5. Thermal Analysis

The thermal decomposition of compounds **1**–**8** was studied by means of differential scanning calorimetry (DSC) and thermogravimetry (TGA) under a flow (20 mL/min) of artificial air (O_2_, (20.9 ± 0.5) vol %; N_2_, (79.1 ± 0.5) vol %; CH_4_, CO, CO_2_, <0.005 vol %). The thermogravimetric measurements were performed on TG 209 F1 instrument in alundum crucibles at a heating rate of 10 °C/min in the range of 25–950 °C. The differential scanning calorimetry studies were carried out on DSC 204 F1 instrument in aluminum cells at a heating rate of 10 °C/min in the range of 25–550 °C. The weight of the samples was 10 to 15 mg (TGA), or 2 to 4 mg (DSC). The thermobalance was calibrated using the phase transition points of standard compounds. The calorimeter was calibrated by temperature (based on the phase transition parameters of standard compounds (C_6_H_12_, Hg, Ga, benzoic acid, KNO_3_, In, Sn, Bi, CsCl of 99.99% purity)) and by phase transition enthalpies according to ISO 11357-1. Data of thermal analysis methods were analyzed according to ISO 11357-1, ISO 11357-2, ISO 11358, and ASTM E 1269-95 standards using the NETZSCH Proteus Thermal Analysis software package.

## 4. Conclusions

In summary, upon crystallization of the products of exchange reactions between 3d-metal salts and potassium cymantrenecarboxylate in the presence of corresponding ligands, we obtained a variety of novel mononuclear complexes. Crystallization in aqueous-organic media led to formation of isostructural compounds consisting of isolated hydrated ions and lattice water molecules [M(H_2_O)_6_](CymCO_2_)_2_·4H_2_O (M = Co, Ni, Zn), while in organic media, in the presence of bipyridyl and imidazole, a number of complexes including these N-bases as ligands was obtained. In the hydrated cymantrenecarboxylates, as well as in the complexes [Co(CymCO_2_)_2_(bpy)_2_]·2PhMe, [Ni(CymCO_2_)(bpy)_2_(H_2_O)][CymCO_2_]·0.5MePh·2H_2_O and [Cu(CymCO_2_)_2_(bpy)(H_2_O)], the transition metal ion has an octahedral environment; the [Co(CymCO_2_)_2_(imz)_2_] complex has a distorted tetrahedral structure and the [Cu(CymCO_2_)_2_(imz)_2_] complex has a square one. In the [Cu(CymCO_2_)_2_(bpy)(H_2_O)] complex, the coordination polyhedron is a square pyramid. Magnetic properties of the cobalt complexes were characterized; it was shown that they exhibit the properties of single-molecule magnets when an external magnetic field is applied. Solid-state thermolysis of the complexes in the air atmosphere in the range of 25–950 °C was studied; it was shown that its specificity is determined by the nature of additional ligands. Complexes with the imidazole ligand are the most thermally stable. It was found that the complexes of cobalt, zinc, and nickel can serve as single-source precursors for tetragonal spinels MMn_2_O_4_ (M = Co, Zn) and a cubic spinel NiMn_2_O_4_, respectively, under thermolysis in air.

## Figures and Tables

**Table 1:** The best-fit parameters of the χT(T) dependences for complexes **1**, **4**, and **5,** calculated using PHI [42].

Parameter	1	4	5
*g_x_*	2.6 ± 0.8	2.3 ± 0.1	2.98 ± 0.2
*g_y_*	2.1 ± 0.9	1.9 ± 0.1	2.3 ± 0.3
*g_z_*	2.657 ± 0.006	2.29 ± 0.01	2.777 ± 0.002
*D*, cm^−1^	−75 ± 2	−14.5 ± 0.6	−65.5 ± 0.5
TIP, cm^−1^	-	8 × 10^−4^ (fixed)	-
Residual, %	99.689	99.666	99.986

**Table 2:** Relaxation parameters and SMM characteristic values for the Co complexes **1**, **4**, and **5**. *R* is the correlation coefficient.

Complex	1	4	5
Field, Oe	2500	1500	1000
Temperature range, K	7–7.2	2–3	3.5–4.5
Δ*E/k_B_*, K	44	13	10
τ_0_, s	4 × 10^−8^	3 × 10^−7^	3.0 × 10^−6^
Temperature range, K	2–7.2	2–3	2–4.5
*C*, K^−*nRaman*^·s^−1^	3.5	135	336
*n_Raman_*	4.7	5.4	3.04
*A*, K^−1^Oe^−4^s^−1^	9.9 × 10^−12^	-	1.91 × 10^−9^
*R* ^2^	0.99937	0.99888	0.99994

## Data Availability

CCDC 1450984-1450986 contain the supplementary crystallographic data for compounds for **1**–**3**, and CCDC 2129830-2129833 for compounds **4**–**8**, respectively. These data can be obtained free of charge via http://www.ccdc.cam.ac.uk/conts/retrieving.html (accessed on 20 December 2021), or from the Cambridge Crystallographic Data Centre, 12 Union Road, Cambridge CB2 1EZ, UK; fax: (+44) 1223-336-033; or e-mail: deposit@ccdc.cam.ac.uk.

## Supplementary Materials

The following supporting information can be downloaded. Table S1: Selected bond lengths [Å] and angles [°] for complexes **1**–**8**; Table S2: H-bonds in the structures of compounds **1**–**4** and **6**–**8** [lengths, Å and angles, °]; Table S3: Structurally characterized mononuclear cymantrenecarboxylates of *d*-metals; Table S4: Crystal data and structure refinement for complexes **1**–**8**; Figure S1: Frequency dependencies of the real, *χ*′ and imaginary, *χ*″ components of dynamic magnetic susceptibility for complex 1 at T = 2 K under various dc magnetic fields (Oe); Figure S2: Frequency dependencies of the real, *χ*′ and imaginary, *χ*″ components of dynamic magnetic susceptibility for complex **4** at T = 2 K under various dc magnetic fields (Oe); Figure S3: Frequency dependencies of the real, *χ*′ and imaginary, *χ*″ components of dynamic magnetic susceptibility for complex **5** at T = 2 K under various dc magnetic fields (Oe); Figure S4: Frequency dependencies of the real (*χ*′) and imaginary (*χ*″) parts of the ac susceptibility for complex **1** at 2500 Oe (2–7.2 K); Figure S5: Frequency dependencies of the real (*χ*′) and imaginary (*χ*″) parts of the ac susceptibility for complex **4** at 1500 Oe (2–3 K); Figure S6: Frequency dependencies of the real (*χ*′) and imaginary (*χ*″) parts of the ac susceptibility for complex **5** at 1000 Oe (2–4.5 K); Figure S7: Powder X-ray diffraction patterns of solid oxidative thermolysis products obtained from the complexes **1**, **3**, **4**, and **5**. Vertical sticks represent the X-ray diffraction patterns of tetragonal spinel AB_2_O_4_; Figure S8: Powder X-ray diffraction patterns of solid oxidative thermolysis products obtained from Ni complexes **2** and **6**. Vertical sticks represent the X-ray diffraction patterns of cubic spinel AB_2_O_4_; Figure S9: Powder X-ray diffraction patterns of solid oxidative thermolysis products obtained from Cu complexes **7** and **8**. Vertical sticks represent the X-ray diffraction patterns of cubic spinel AB_2_O_4_ and CuO.

## Sample Availability

Samples of the compounds reported in this article are not available from the authors.
