# Supplementary material for: Mononuclear Transition Metal Cymantrenecarboxylates as Precursors for Spinel-Type Manganites
Source: Molecules. 2022 Feb 6;27(3):1082. doi: 10.3390/molecules27031082 (PMC8838078; doi:10.3390/molecules27031082)
Supplement: Supplementary file 1 [file molecules-27-01082-s001.zip › molecules-1552484-supplementary.pdf]

Mononuclear transition metal cymantrenecarboxylates as precursors for spinel-type manganites

Pavel S. Koroteev, Andrey B. Ilyukhin, Andrey V. Gavrikov, Konstantin A. Babeshkin, Nikolay N. Efimov

N.S. Kurnakov Institute of General and Inorganic Chemistry, Russian Academy of Sciences, Leninsky prosp. 31, 119991 Moscow, GSP-1, Russian Federation, pskoroteev@list.ru

SUPPLEMENTARY MATERIALS

Table S1. Selected bond lengths [Å] and angles [°] for complexes **1-8**.

	**1** (M=Co)	**2** (M=Ni)	**3** (M=Zn)
M(1)-O(11)	2.1212(10)	2.0828(11)	2.169(2)
M(1)-O(12)	2.1319(11)	2.0896(11)	2.173(2)
M(1)-O(13)	2.0883(11)	2.0474(11)	2.121(2)
M(1)-O(14)	2.0830(12)	2.0469(11)	2.115(2)
M(1)-O(15)	2.0704(12)	2.0467(12)	2.120(2)
M(1)-O(16)	2.0752(11)	2.0552(12)	2.129(2)
Mn(1)-C(2)	2.1384(14)	2.1404(14)	2.143(3)
Mn(1)-C(3)	2.1430(16)	2.1401(15)	2.145(3)
Mn(1)-C(4)	2.1437(16)	2.1414(16)	2.140(3)
Mn(1)-C(5)	2.1439(16)	2.1433(15)	2.143(3)
Mn(1)-C(6)	2.1415(15)	2.1394(14)	2.135(3)
Mn(1)-C(13)	1.7971(17)	1.7961(17)	1.794(3)
Mn(1)-C(14)	1.7867(18)	1.7915(18)	1.790(4)
Mn(1)-C(15)	1.7936(18)	1.7920(17)	1.792(3)
Mn(2)-C(8)	2.1336(14)	2.1328(13)	2.129(3)
Mn(2)-C(9)	2.1395(15)	2.1405(14)	2.139(3)
Mn(2)-C(10)	2.1526(16)	2.1526(15)	2.153(3)
Mn(2)-C(11)	2.1543(15)	2.1554(15)	2.150(3)
Mn(2)-C(12)	2.1459(15)	2.1438(14)	2.143(3)
Mn(2)-C(16)	1.8045(16)	1.8056(16)	1.804(3)
Mn(2)-C(17)	1.8009(18)	1.8020(17)	1.804(3)
Mn(2)-C(18)	1.7886(17)	1.7872(17)	1.785(3)
O(11)-M(1)-O(12)	86.35(4)	87.96(4)	85.71(8)

O(11)-M(1)-O(13)	89.02(5)	89.33(5)	90.09(8)
O(11)-M(1)-O(14)	95.87(5)	94.39(5)	96.91(8)
O(11)-M(1)-O(15)	89.94(5)	89.51(5)	89.76(9)
O(11)-M(1)-O(16)	174.27(5)	176.40(5)	172.46(9)
O(12)-M(1)-O(13)	96.50(5)	96.10(5)	97.05(8)
O(12)-M(1)-O(14)	89.72(5)	90.49(5)	90.59(8)
O(12)-M(1)-O(15)	174.81(5)	176.24(5)	174.24(9)
O(12)-M(1)-O(16)	88.86(5)	89.27(5)	87.54(9)
O(13)-M(1)-O(14)	172.33(5)	172.54(5)	170.02(8)
O(13)-M(1)-O(15)	87.05(5)	86.64(5)	86.50(9)
O(13)-M(1)-O(16)	88.41(5)	88.68(5)	87.40(8)
O(14)-M(1)-O(15)	87.05(5)	86.94(5)	86.42(8)
O(14)-M(1)-O(16)	87.24(5)	87.93(5)	86.52(9)
O(15)-M(1)-O(16)	95.04(6)	93.38(6)	97.17(10)

4

Co(1)-O(1)	1.9776(19)
Co(1)-O(6)	1.9586(19)
Co(1)-N(1)	2.011(2)
Co(1)-N(3)	2.019(2)
Mn(1)-C(2)	2.120(3)
Mn(1)-C(3)	2.128(3)
Mn(1)-C(4)	2.137(3)
Mn(1)-C(5)	2.144(3)
Mn(1)-C(6)	2.136(3)
Mn(1)-C(7)	1.793(3)
Mn(1)-C(8)	1.787(3)
Mn(1)-C(9)	1.787(3)
Mn(2)-C(11)	2.128(3)
Mn(2)-C(12)	2.136(3)
Mn(2)-C(13)	2.136(3)
Mn(2)-C(14)	2.135(3)
Mn(2)-C(15)	2.132(3)
Mn(2)-C(16)	1.799(3)
Mn(2)-C(17)	1.795(3)
Mn(2)-C(18)	1.790(4)

O(6)-Co(1)-O(1)	129.77(9)
O(6)-Co(1)-N(1)	106.46(9)
O(1)-Co(1)-N(1)	97.44(9)

O(6)-Co(1)-N(3)	99.51(9)
O(1)-Co(1)-N(3)	110.18(9)
N(1)-Co(1)-N(3)	113.97(10)

5

Co(1)-O(1)	2.0374(16)
Co(1)-N(1)	2.1512(19)
Co(1)-N(2)	2.1519(18)
Mn(1)-C(2)	2.147(2)
Mn(1)-C(3)	2.147(2)
Mn(1)-C(4)	2.134(3)
Mn(1)-C(5)	2.140(3)
Mn(1)-C(6)	2.145(2)
Mn(1)-C(7)	1.791(3)
Mn(1)-C(8)	1.786(3)
Mn(1)-C(9)	1.795(3)

O(1)#1-Co(1)-O(1)	89.35(9)
O(1)#1-Co(1)-N(1)	162.78(7)
O(1)-Co(1)-N(1)	91.87(7)
O(1)#1-Co(1)-N(1)#1	91.87(7)
O(1)-Co(1)-N(1)#1	162.78(7)
N(1)-Co(1)-N(1)#1	92.02(10)
O(1)#1-Co(1)-N(2)#1	101.18(7)
O(1)-Co(1)-N(2)#1	87.47(7)
N(1)-Co(1)-N(2)#1	96.03(7)
N(1)#1-Co(1)-N(2)#1	75.44(7)
O(1)#1-Co(1)-N(2)	87.47(7)
O(1)-Co(1)-N(2)	101.18(7)
N(1)-Co(1)-N(2)	75.44(7)
N(1)#1-Co(1)-N(2)	96.03(7)
N(2)#1-Co(1)-N(2)	167.91(10)

#1 -x+1, y, -z+1/2

6

Ni(1)-O(1)	2.067(2)
Ni(1)-O(11)	2.072(3)
Ni(1)-N(1)	2.076(3)
Ni(1)-N(2)	2.097(3)

Ni(1)-N(3)	2.089(3)
Ni(1)-N(4)	2.079(3)
Mn(1)-C(2)	2.130(4)
Mn(1)-C(3)	2.148(4)
Mn(1)-C(4)	2.152(4)
Mn(1)-C(5)	2.146(4)
Mn(1)-C(6)	2.131(4)
Mn(1)-C(7)	1.787(5)
Mn(1)-C(8)	1.788(4)
Mn(1)-C(9)	1.785(4)
Mn(2)-C(11)	2.153(4)
Mn(2)-C(12)	2.140(4)
Mn(2)-C(13)	2.136(4)
Mn(2)-C(14)	2.137(4)
Mn(2)-C(15)	2.144(3)
Mn(2)-C(16)	1.790(4)
Mn(2)-C(17)	1.795(5)
Mn(2)-C(18)	1.776(4)

O(1)-Ni(1)-O(11)	91.91(10)
O(1)-Ni(1)-N(1)	87.93(11)
O(11)-Ni(1)-N(1)	167.18(11)
O(1)-Ni(1)-N(4)	91.54(11)
O(11)-Ni(1)-N(4)	94.04(11)
N(1)-Ni(1)-N(4)	98.78(11)
O(1)-Ni(1)-N(3)	169.64(11)
O(11)-Ni(1)-N(3)	89.88(11)
N(1)-Ni(1)-N(3)	92.57(11)
N(4)-Ni(1)-N(3)	78.15(12)
O(1)-Ni(1)-N(2)	89.37(11)
O(11)-Ni(1)-N(2)	88.89(11)
N(1)-Ni(1)-N(2)	78.29(12)
N(4)-Ni(1)-N(2)	176.90(12)
N(3)-Ni(1)-N(2)	100.87(11)

7

Cu(1)-O(1)	1.938(8)
Cu(1)-N(1)	1.971(10)
Mn(1)-C(2)	2.132(11)
Mn(1)-C(3)	2.125(12)

Mn(1)-C(4)	2.120(12)
Mn(1)-C(5)	2.123(12)
Mn(1)-C(6)	2.119(13)
Mn(1)-C(7)	1.794(17)
Mn(1)-C(8)	1.768(18)
Mn(1)-C(9)	1.782(16)

O(1)-Cu(1)-O(1)#1	180.0
O(1)-Cu(1)-N(1)#1	89.1(4)
O(1)#1-Cu(1)-N(1)#1	90.9(4)
O(1)-Cu(1)-N(1)	90.8(4)
O(1)#1-Cu(1)-N(1)	89.2(4)
N(1)#1-Cu(1)-N(1)	180.0

#1 -x+1, -y, -z+1

8

Cu(1)-O(1)	1.946(3)
Cu(1)-O(6)	1.983(3)
Cu(1)-O(11)	2.215(3)
Cu(1)-N(1)	2.021(4)
Cu(1)-N(2)	2.017(4)
Mn(1)-C(2)	2.125(4)
Mn(1)-C(3)	2.130(5)
Mn(1)-C(4)	2.156(5)
Mn(1)-C(5)	2.156(5)
Mn(1)-C(6)	2.141(5)
Mn(1)-C(7)	1.792(5)
Mn(1)-C(8)	1.785(5)
Mn(1)-C(9)	1.791(5)
Mn(2)-C(11)	2.134(5)
Mn(2)-C(12)	2.148(5)
Mn(2)-C(13)	2.155(5)
Mn(2)-C(14)	2.143(5)
Mn(2)-C(15)	2.130(5)
Mn(2)-C(16)	1.801(6)
Mn(2)-C(17)	1.803(5)
Mn(2)-C(18)	1.792(6)

O(1)-Cu(1)-O(6)	91.29(14)
O(1)-Cu(1)-N(2)	171.93(15)
O(6)-Cu(1)-N(2)	92.42(15)
O(1)-Cu(1)-N(1)	95.25(15)
O(6)-Cu(1)-N(1)	167.71(15)
N(2)-Cu(1)-N(1)	79.84(16)
O(1)-Cu(1)-O(11)	90.37(14)
O(6)-Cu(1)-O(11)	93.75(14)
N(2)-Cu(1)-O(11)	96.53(14)
N(1)-Cu(1)-O(11)	96.59(14)

Table S2. H-bonds in the structures of compounds **1-4** and **6-8** [lengths, Å and angles, °].

D-H...A	d(D-H)	d(H...A)	d(D...A)	<(DHA)
		**1**		
O(11)-H(1)...O(17)	0.71(2)	2.03(2)	2.7334(16)	169(2)
O(11)-H(2)...O(2) (-x, -y, -z)	0.82(2)	1.91(2)	2.7227(16)	179(2)
O(12)-H(3)...O(1) (-x, -y, -z)	0.83(3)	1.89(3)	2.6993(15)	163(3)
O(12)-H(4)...O(19) (-x+1, -y, -z)	0.87(2)	1.88(2)	2.7427(17)	170(2)
O(13)-H(5)...O(17) (x+1, y, z)	0.84(2)	1.89(2)	2.7305(16)	172(2)
O(13)-H(6)...O(4) (x+1, y, z)	0.79(2)	2.05(2)	2.8318(15)	171(2)
O(14)-H(7)...O(3)	0.80(2)	1.97(2)	2.7682(16)	176(2)
O(14)-H(8)...O(19) (-x, -y, -z)	0.71(2)	2.00(2)	2.7102(16)	171(3)
O(15)-H(9)...O(11) (-x, -y+1, -z)	0.74(2)	2.16(2)	2.8870(16)	167(2)
O(15)-H(10)...O(4)	0.79(3)	1.96(3)	2.7444(17)	174(3)
O(16)-H(11)...O(20)	0.79(2)	1.95(2)	2.7312(18)	170(2)
O(16)-H(12)...O(3) (x+1, y, z)	0.80(2)	1.96(2)	2.7594(17)	174.6(19)
O(17)-H(13)...O(2) (-x-1, -y, -z)	0.81(2)	2.08(2)	2.8575(16)	160(2)
O(17)-H(14)...O(4) (-x-1, -y+1, -z)	0.77(3)	1.94(3)	2.6998(15)	169(3)
O(18)-H(15)...O(3) (x+1, y, z)	0.77(2)	1.98(2)	2.7496(16)	172(2)
O(18)-H(16)...O(1)	0.85(3)	1.98(3)	2.8178(17)	172(3)
O(19)-H(17)...O(20) (x+1, y, z)	0.83(2)	1.87(2)	2.6826(18)	164(2)
O(19)-H(18)...O(12) (x+1, y, z)	0.78(2)	2.11(2)	2.8726(17)	166(2)
O(20)-H(19)...O(18) (x-1, y, z)	0.72(3)	1.97(3)	2.668(2)	164(3)
O(20)-H(20)...O(1)	0.83(3)	2.00(3)	2.8028(18)	162(2)
		**2**		
O(11)-H(1)...O(17)	0.74(2)	2.01(2)	2.7278(15)	166(2)
O(11)-H(2)...O(2) (-x, -y, -z)	0.81(2)	1.92(2)	2.7260(15)	177(2)
O(12)-H(3)...O(1) (-x, -y, -z)	0.85(2)	1.90(2)	2.7337(16)	169(2)

O(12)-H(4)...O(19) (-x+1, -y, -z)	0.78(3)	1.94(3)	2.6999(16)	163(2)
O(13)-H(5)...O(17) (x+1, y, z)	0.73(2)	1.99(2)	2.7174(16)	174(2)
O(13)-H(6)...O(4) (x+1, y, z)	0.80(2)	2.03(2)	2.8245(15)	174(2)
O(14)-H(7)...O(3)	0.73(2)	1.99(2)	2.7044(16)	169(2)
O(14)-H(8)...O(19) (-x, -y, -z)	0.85(2)	1.92(2)	2.7688(16)	172(2)
O(15)-H(9)...O(11) (-x, -y+1, -z)	0.76(3)	1.99(3)	2.7474(16)	176(2)
O(15)-H(10)...O(4)	0.80(2)	2.10(2)	2.8846(16)	167(2)
O(16)-H(11)...O(20)	0.79(2)	1.98(2)	2.7739(16)	176.6(19)
O(16)-H(12)...O(3) (x+1, y, z)	0.78(3)	1.97(3)	2.7334(17)	165(3)
O(17)-H(13)...O(2) (-x-1, -y, -z)	0.80(2)	2.10(2)	2.8505(15)	157(2)
O(17)-H(14)...O(4) (-x-1, -y+1, -z)	0.78(2)	1.93(2)	2.6952(15)	170(2)
O(18)-H(15)...O(3) (x+1, y, z)	0.73(3)	2.02(3)	2.7471(17)	174(3)
O(18)-H(16)...O(1)	0.82(2)	1.99(3)	2.8102(17)	172(2)
O(19)-H(17)...O(20) (x+1, y, z)	0.83(3)	1.84(3)	2.6696(18)	174(3)
O(19)-H(18)...O(12) (x+1, y, z)	0.75(3)	2.17(3)	2.8894(17)	160(2)
O(20)-H(19)...O(18) (x-1, y, z)	0.84(3)	2.02(3)	2.8009(17)	155(2)
O(20)-H(20)...O(1)	0.71(3)	1.97(3)	2.6649(19)	168(3)
		**3**		
O(11)-H(1)...O(17)	0.90	1.83	2.717(3)	169
O(11)-H(2)...O(2) (-x, -y, -z)	0.90	1.88	2.742(3)	161
O(12)-H(3)...O(1) (-x, -y, -z)	0.90	1.84	2.737(3)	171
O(12)-H(4)...O(19) (-x+1, -y, -z)	0.90	1.80	2.693(3)	170
O(13)-H(5)...O(17) (x+1, y, z)	0.90	1.92	2.814(3)	175
O(13)-H(6)...O(4) (x+1, y, z)	0.90	1.84	2.734(3)	174
O(14)-H(7)...O(3)	0.90	1.86	2.753(3)	171
O(14)-H(8)...O(19) (-x, -y, -z)	0.90	1.81	2.696(3)	168
O(15)-H(9)...O(11) (-x, -y+1, -z)	0.90	1.85	2.745(3)	177
O(15)-H(10)...O(4)	0.90	2.05	2.887(3)	154
O(16)-H(11)...O(20)	0.90	1.84	2.723(3)	168
O(16)-H(12)...O(3) (x+1, y, z)	0.90	1.87	2.762(3)	174
O(17)-H(13)...O(2) (-x-1, -y, -z)	0.90	1.84	2.701(3)	159
O(17)-H(14)...O(4) (-x-1, -y+1, -z)	0.90	2.00	2.855(3)	157
O(18)-H(15)...O(3) (x+1, y, z)	0.90	1.87	2.741(3)	162
O(18)-H(16)...O(1)	0.90	1.91	2.807(3)	176
O(19)-H(17)...O(20) (x+1, y, z)	0.90	2.00	2.875(3)	162
O(19)-H(18)...O(12) (x+1, y, z)	0.90	1.79	2.680(3)	169
O(20)-H(19)...O(18) (x-1, y, z)	0.90	1.80	2.673(3)	164
O(20)-H(20)...O(1)	0.90	1.97	2.807(3)	153
		**4**		
N(2)-H(1)...O(7) (-x, -y+1, -z)	0.91(3)	1.84(3)	2.736(3)	168(3)
N(4)-H(2)...O(2) (-x+1/2, y+1/2, -z+1/2)	0.94(4)	1.80(4)	2.722(3)	164(3)
		**6**		

O(11)-H(1)...O(2)	0.85(4)	1.79(4)	2.620(4)	165(4)
O(11)-H(2)...O(7)	0.78(4)	1.89(4)	2.657(4)	173(4)
O(12)-H(3)...O(13)	0.92(4)	1.86(4)	2.762(4)	165(4)
O(12)-H(4)...O(6)	0.78(4)	1.96(4)	2.723(4)	169(5)
O(13)-H(5)...O(7) (x, y, z-1)	0.73(4)	2.10(4)	2.825(4)	173(5)
O(13)-H(6)...O(12) (x, -y+1/2, z-1/2)	0.88(4)	1.82(4)	2.691(4)	168(4)
	**7**			
N(2)-H(2A)...O(2) (x, -y+1/2, z-1/2)	0.86	1.93	2.79(2)	158
	**8**			
O(11)-H(1)...O(7) (x, y+1, z)	0.77	1.96	2.714(5)	166
O(11)-H(2)...O(2) (x, y+1, z)	0.89	1.99	2.758(5)	144

Table S3. Structurally characterized mononuclear cymantrenecarboxylates of *d*-metals (CSD version 5.42 updates (Sep 2021)*).

AFATUZ. A.A.Grineva, M.A.Uvarova, R.R.Datchuk, S.E.Nefedov. Russian J.Inorg.Chem., 63, 610, 2018.

AFAVAH. A.A.Grineva, M.A.Uvarova, R.R.Datchuk, S.E.Nefedov. Russian J.Inorg.Chem., 63, 610, 2018.

AFAVEL. A.A.Grineva, M.A.Uvarova, R.R.Datchuk, S.E.Nefedov. Russian J.Inorg.Chem., 63, 610, 2018.

AFAVIP. A.A.Grineva, M.A.Uvarova, R.R.Datchuk, S.E.Nefedov. Russian J.Inorg.Chem., 63, 610, 2018.

AFEJED. A.A.Grineva, M.A.Uvarova, R.R.Datchuk, S.E.Nefedov. Russian J.Inorg.Chem., 63, 610, 2018.

AJUBUE. S.S.Shapovalov, A.A.Pasynsky, I.V.Skabitsky, T.A.Krishtop, Zh.V.Dobrokhotova. Russian J.Coord.Chem., 40, 77, 2014.

AJUCIT. S.S.Shapovalov, A.A.Pasynsky, I.V.Skabitsky, T.A.Krishtop, Zh.V.Dobrokhotova. Russian J.Coord.Chem., 40, 77, 2014.

HULWAP. A.V.Gavrikov, A.B.Ilyukhin, P.S.Koroteev. CrystEngComm, 22, 2895, 2020.

HULWET. A.V.Gavrikov, A.B.Ilyukhin, P.S.Koroteev. CrystEngComm, 22, 2895, 2020.

HULWIX. A.V.Gavrikov, A.B.Ilyukhin, P.S.Koroteev. CrystEngComm, 22, 2895, 2020.

MUYBIT. M.A.Uvarova, A.A.Ageshina, S.E.Nefedov. Russian J.Inorg.Chem., 60, 1210, 2015.

MUYBOZ. M.A.Uvarova, A.A.Ageshina, S.E.Nefedov. Russian J.Inorg.Chem., 60, 1210, 2015.

MUYCAM. M.A.Uvarova, A.A.Ageshina, S.E.Nefedov. Russian J.Inorg.Chem., 60, 1218, 2015.

MUYJUN. M.A.Uvarova, S.E.Nefedov. Russian J.Inorg.Chem., 60, 1074, 2015.

MUYKAU. M.A.Uvarova, S.E.Nefedov. Russian J.Inorg.Chem., 60, 1074, 2015.

MUYKUO. A.A.Ageshina, M.A.Uvarova, S.E.Nefedov. Russian J.Inorg.Chem., 60, 1085, 2015.

OTEYEU. A.V.Gavrikov, E.V.Belova, A.B.Ilyukhin, P.S.Koroteev, A.A.Sadovnikov. Appl.Organomet.Chem., 35, e6190, 2021.

OTEZAR. A.V.Gavrikov, E.V.Belova, A.B.Ilyukhin, P.S.Koroteev, A.A.Sadovnikov. Appl.Organomet.Chem., 35, e6190, 2021.

OTEZOF. A.V.Gavrikov, E.V.Belova, A.B.Ilyukhin, P.S.Koroteev, A.A.Sadovnikov. Appl.Organomet.Chem., 35, e6190, 2021.

---

* Groom C. R., Bruno I. J., Lightfoot M. P., Ward S. C. The Cambridge Structural Database. *Acta Crystallographica Section B*. **2016**. V.*72*. №2. P. 171-179.

Table S4. Crystal data and structure refinement for complexes **1-8**.

Identification code	**1**	**2**	**3**	**4**
Empirical formula	$C_{18}H_{28}CoMn_2O_{20}$	$C_{18}H_{28}Mn_2NiO_{20}$	$C_{18}H_{28}Mn_2O_{20}Zn$	$C_{24}H_{16}CoMn_2N_4O_{10}$
Formula weight	733.21	732.99	739.65	689.22
Temperature, K	173(2)	173(2)	173(2)	150(2)
Wavelength, Å	0.71073	0.71073	0.71073	0.71073
Crystal system	Triclinic	Triclinic	Triclinic	Monoclinic
Space group	P-1	P-1	P-1	$P2_1/n$
a, Å	6.4016(5)	6.3826(2)	6.4041(2)	13.9056(7)
b, Å	12.6294(9)	12.6104(3)	12.6715(5)	7.5358(4)
c, Å	18.5659(13)	18.5067(4)	18.6201(7)	26.8049(14)
α, °	93.005(3)	93.1140(10)	93.1270(10)	90
β, °	96.058(3)	95.9200(10)	95.7570(10)	103.165(2)
γ, °	103.604(3)	103.6810(10)	103.9860(10)	90
Volume, $Å^3$	1446.12(18)	1434.79(6)	1453.96(9)	2735.1(2)
Z	2	2	2	4
D (calc), Mg/m^3	1.684	1.697	1.689	1.674
μ, mm^{-1}	1.511	1.601	1.757	1.571
F(000)	746	748	752	1380
Crystal size, mm	0.36 x 0.24 x 0.10	0.40 x 0.32 x 0.10	0.32 x 0.3 x 0.08	0.28 x 0.24 x 0.18
θ range, °	2.071, 33.256	2.220, 32.940	2.206, 33.002	2.478, 28.334
Index ranges	-9<=h<=9<br>-18<=k<=18<br>-28<=l<=28	-9<=h<=9<br>-18<=k<=18<br>-26<=l<=27	-9<=h<=9<br>-19<=k<=19<br>-28<=l<=28	-18<=h<=18<br>-10<=k<=10<br>-35<=l<=35
Reflections collected	17299	21786	22125	32727
Independent reflections, Rint	9683, 0.0356	9755, 0.0328	9882, 0.0347	6806, 0.0505
Completeness to θ = 25.242°	99.8 %	99.9 %	99.9 %	99.9 %
Absorption correction	Semi-empirical from equivalents	Semi-empirical from equivalents	Semi-empirical from equivalents	Semi-empirical from equivalents
Max,. min. transmission	0.7465, 0.4958	0.7465, 0.5444	0.7465, 0.5488	0.7457, 0.574
Refinement method	Full-matrix least-squares on F^2	Full-matrix least-squares on F^2	Full-matrix least-squares on F^2	Full-matrix least-squares on F^2
Data / restraints / parameters	9683 / 1 / 450	9755 / 0 / 450	9882 / 0 / 370	6806 / 0 / 376
Goodness-of-fit	1.004	1.037	1.121	1.015
R1, wR2 [I>2sigma(I)]	0.0316, 0.0796	0.0314, 0.0789	0.0515, 0.1532	0.0398, 0.0950
R1, wR2 (all data)	0.0409, 0.0835	0.0410, 0.0829	0.0665, 0.1631	0.0611, 0.1048
Largest diff. peak and hole, e.$Å^{-3}$	0.576, -0.413	0.608, -0.421	1.150, -2.073	0.614, -0.356

Identification code	**5**	**6**	**7**	**8**
Empirical formula	$C_{52}H_{40}CoMn_2N_4O_{10}$	$C_{41.50}H_{34}Mn_2N_4NiO_{13}$	$C_{24}H_{16}CuMn_2N_4O_{10}$	$C_{28}H_{18}CuMn_2N_2O_{11}$
Formula weight	1049.69	965.32	693.83	731.86
Temperature, K	150(2)	120(2)	296(2)	120(2)
Wavelength, Å	0.71073	0.71073	0.71073	0.71073
Crystal system	Monoclinic	Monoclinic	Monoclinic	Monoclinic
Space group	C2/c	$P2_1/c$	$P2_1/c$	$P2_1/c$
a, Å	25.0607(13)	12.7133(17)	7.955(4)	15.5777(10)
b, Å	10.7040(5)	44.403(6)	14.956(8)	6.7135(4)
c, Å	18.4501(9)	7.2002(10)	11.228(6)	26.6800(17)
β, °	107.166(2)	101.017(3)	93.383(16)	91.316(2)
Volume, $Å^3$	4728.8(4)	3989.7(9)	1333.5(12)	2789.5(3)
Z	4	4	2	4
D (calc), Mg/m^3	1.474	1.607	1.728	1.743
μ, mm^{-1}	0.937	1.164	1.786	1.714
F(000)	2148	1972	694	1468
Crystal size, mm	0.22 x 0.12 x 0.10	0.26 x 0.08 x 0.02	0.20 x 0.02 x 0.02	0.26 x 0.04 x 0.02
θ range, °	2.084, 27.919	2.135, 26.429	2.271, 25.396	2.291, 30.054
Index ranges	-32<=h<=32<br>-14<=k<=14<br>-24<=l<=24	-15<=h<=13<br>-55<=k<=55<br>-9<=l<=9	-9<=h<=9<br>0<=k<=18<br>-13<=l<=13	-21<=h<=21<br>-9<=k<=9<br>-37<=l<=37
Reflections collected	28267	28019	4851	56077
Independent reflections, Rint	5643, 0.0556	8141, 0.0919	2442, 0.1272	8287, 0.0830
Completeness to θ = 25.242°	100.0 %	99.6 %	100.0 %	99.8 %
Absorption correction	Semi-empirical from equivalents	Semi-empirical from equivalents	Semi-empirical from equivalents	Semi-empirical from equivalents
Max,. min. transmission	0.7456, 0.6283	0.7454, 0.6399	0.7452, 0.5738	0.7456, 0.6561
Refinement method	Full-matrix least-squares on F^2	Full-matrix least-squares on F^2	Full-matrix least-squares on F^2	Full-matrix least-squares on F^2
Data / restraints / parameters	5643 / 42 / 328	8141 / 42 / 605	2442 / 0 / 187	8287 / 0 / 398
Goodness-of-fit	0.965	1.004	1.156	1.086
R1, wR2 [I>2sigma(I)]	0.0397, 0.1092	0.0499, 0.0822	0.1029, 0.2360	0.0550, 0.1522
R1, wR2 (all data)	0.0585, 0.1235	0.1040, 0.0958	0.1885, 0.2673	0.0736, 0.1644
Largest diff. peak and hole, e.$Å^{-3}$	1.015, -0.311	0.535, -0.470	1.925, -0.549	2.098, -0.893

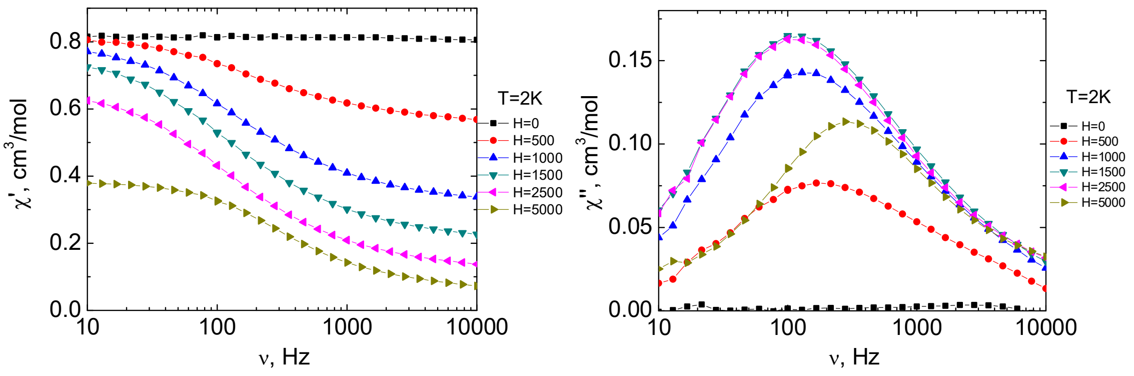

Figure S1. Frequency dependencies of the real, χ' (left) and imaginary, χ'' (right) components of dynamic magnetic susceptibility for complex **1** at T = 2 K under various dc magnetic fields (Oe). Solid lines are visual guides.

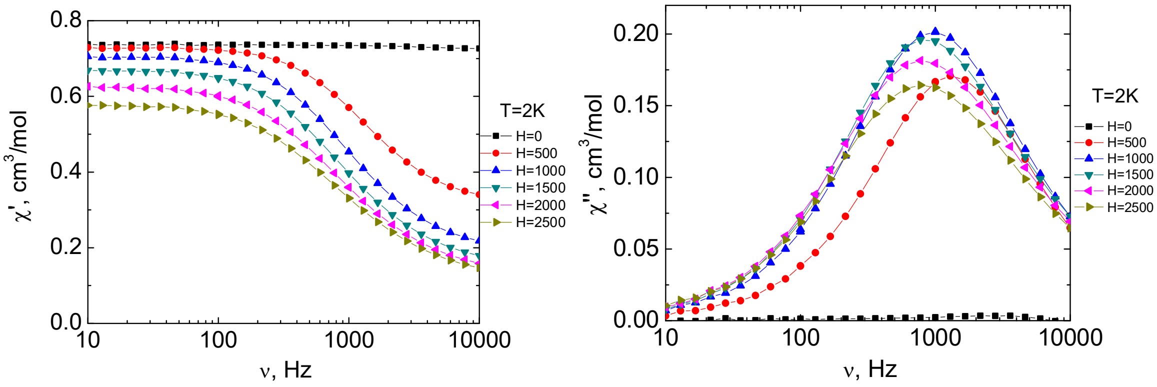

Figure S2. Frequency dependencies of the real, χ' (left) and imaginary, χ'' (right) components of dynamic magnetic susceptibility for complex **4** at T = 2 K under various dc magnetic fields (Oe). Solid lines are visual guides.

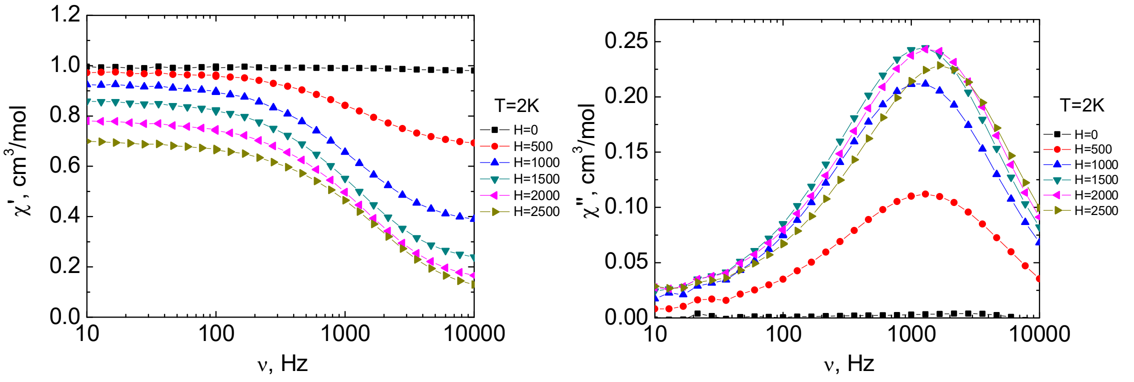

Figure S3. Frequency dependencies of the real, χ' (left) and imaginary, χ'' (right) components of dynamic magnetic susceptibility for complex **5** at T = 2 K under various dc magnetic fields (Oe). Solid lines are visual guides.

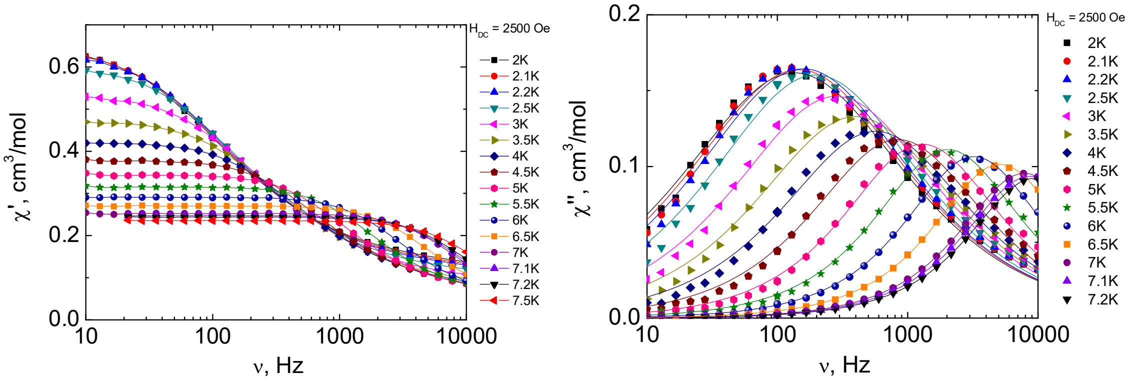

Figure S4. Frequency dependencies of the real (χ', left) and imaginary (χ'', right) parts of the ac susceptibility for complex **1** at 2500 Oe (2-7.2 K). Lines represent visual guides (left) and approximations of the experimental data by the generalized Debye model (right).

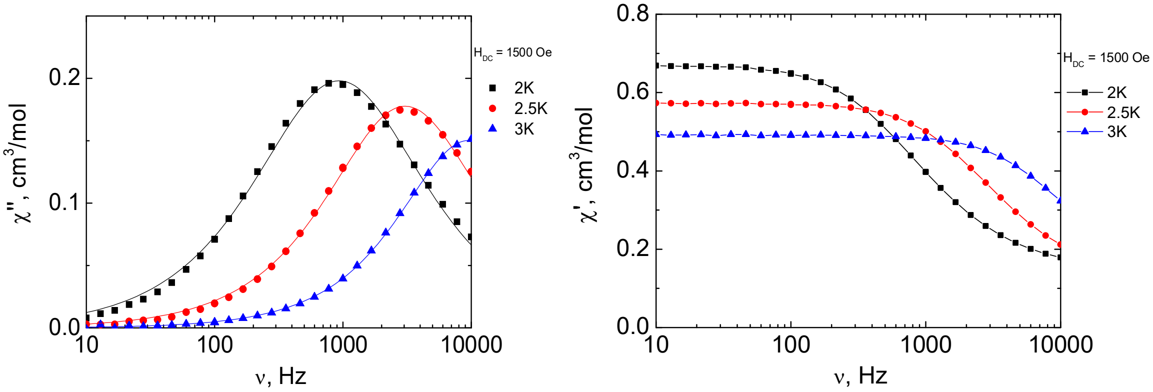

Figure S5. Frequency dependencies of the real (χ', left) and imaginary (χ'', right) parts of the ac susceptibility for complex **4** at 1500 Oe (2-3 K). Lines represent visual guides (left) and approximations of the experimental data by the generalized Debye model (right).

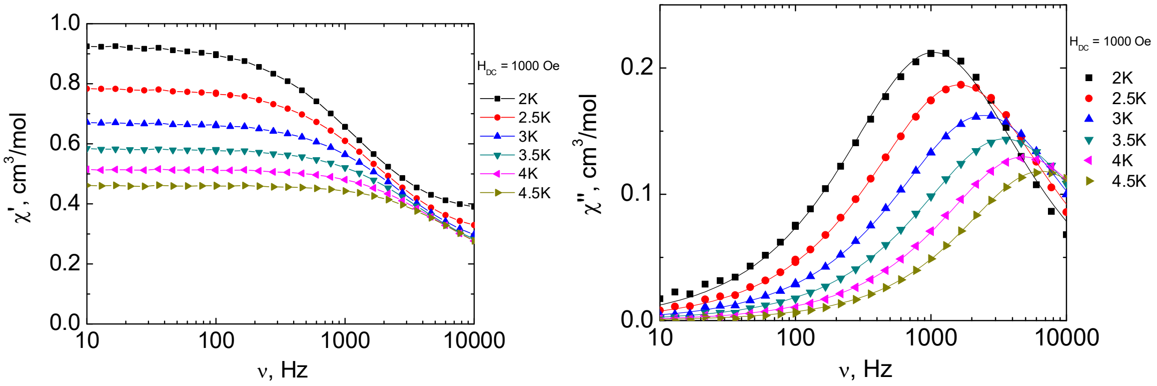

Figure S6. Frequency dependencies of the real (χ', left) and imaginary (χ'', right) parts of the ac susceptibility for complex **5** at 1000 Oe (2-4.5 K). Lines represent visual guides (left) and approximations of the experimental data by the generalized Debye model (right).

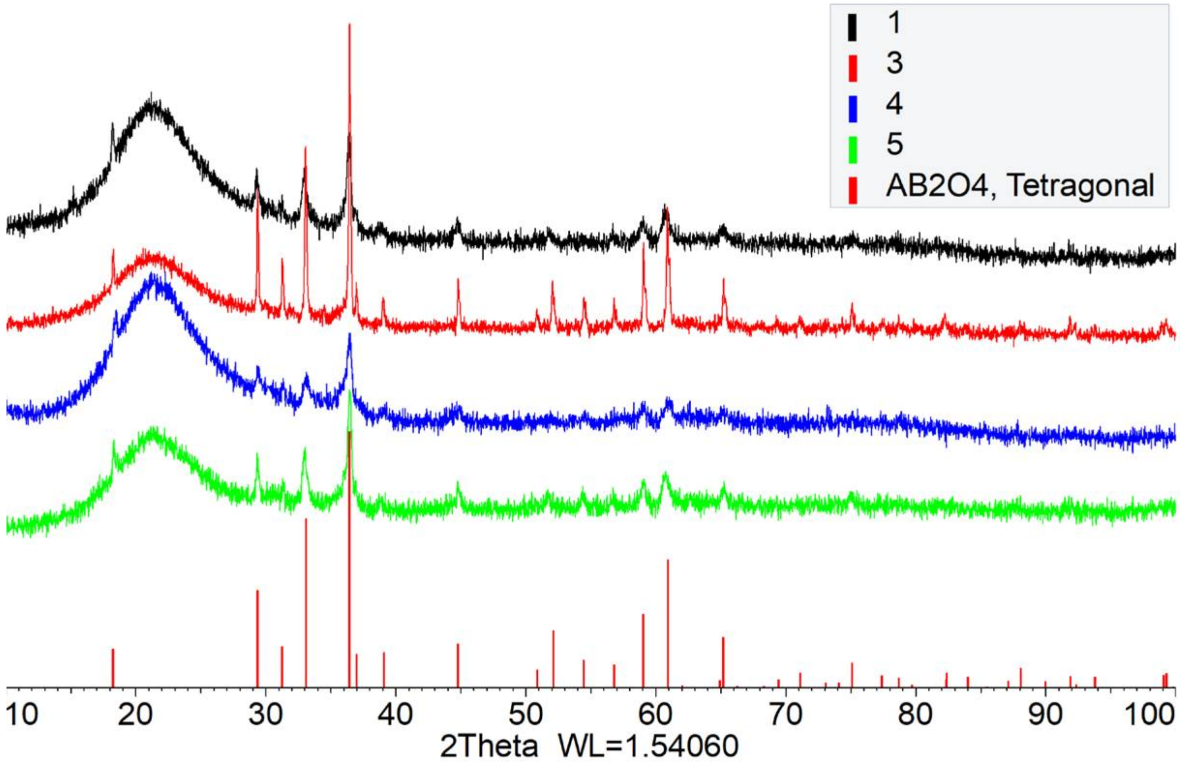

Figure S7. Powder X-ray diffraction patterns of solid oxidative thermolysis products obtained from the complexes **1** (**black** line), **3** (**red** line), **4** (**blue** line), and **5** (**green** line). Vertical sticks represent the X-ray diffraction patterns of tetragonal spinel AB_2O_4.

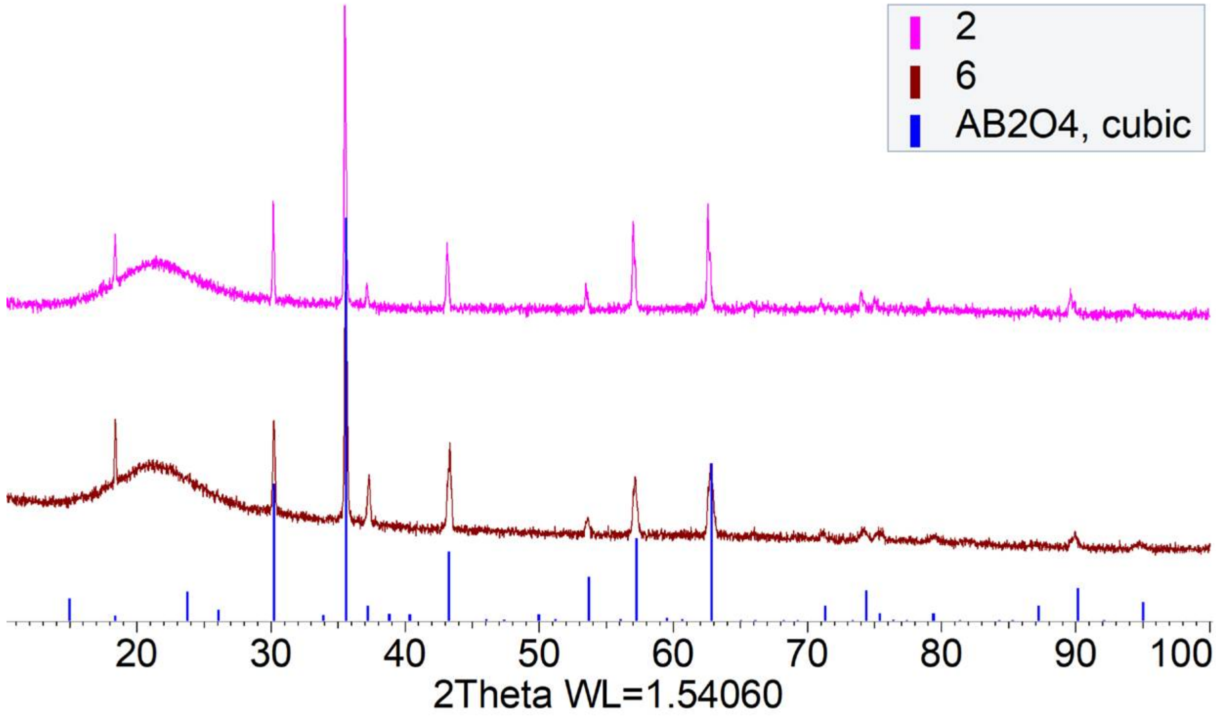

Figure S8. Powder X-ray diffraction patterns of solid oxidative thermolysis products obtained from Ni complexes **2** (**magenta** line) and **6** (**brown** line). Vertical sticks represent the X-ray diffraction patterns of cubic spinel AB_2O_4.

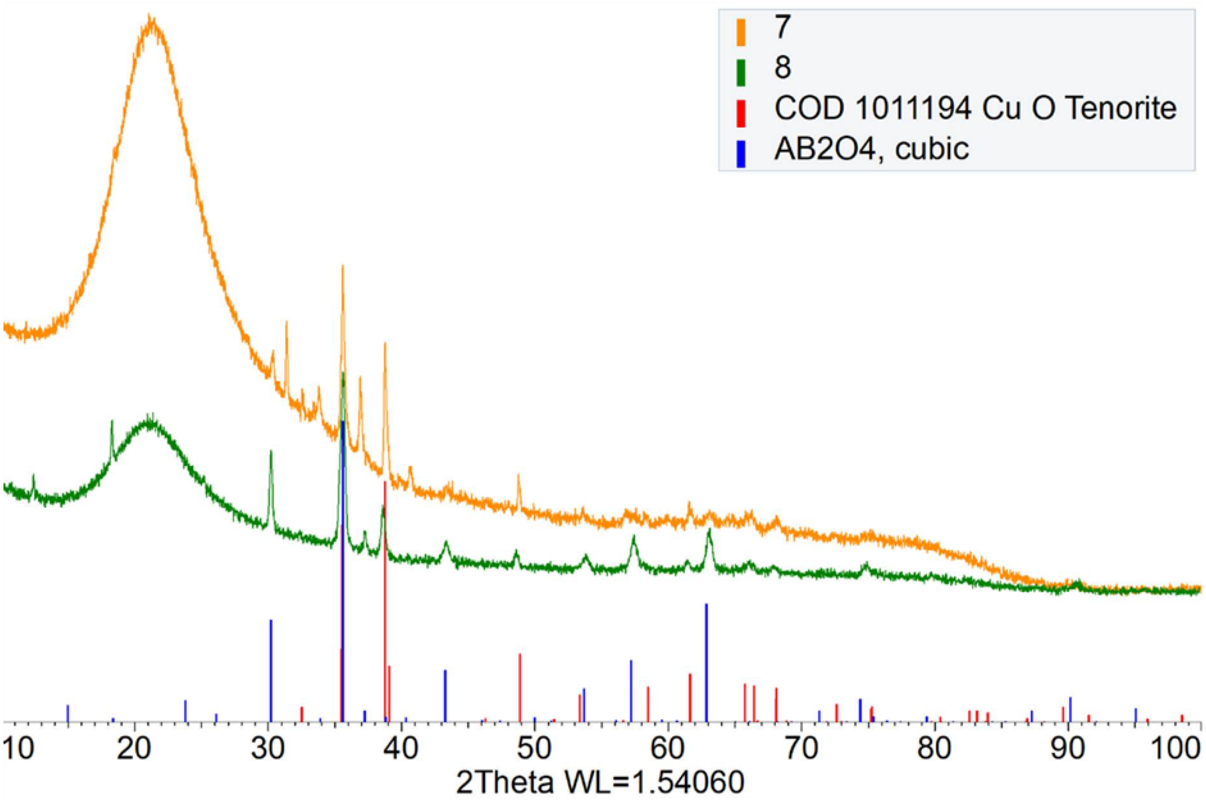

Figure S9. Powder X-ray diffraction patterns of solid oxidative thermolysis products obtained from Cu complexes **7** (**orange** line) and **8** (**green** line). Vertical sticks represent the X-ray diffraction patterns of cubic spinel AB_2O_4 and CuO.
